# Kinetics and mechanism of sequential ring methyl C–H activation in cyclopentadienyl rhodium(iii) complexes†

**DOI:** 10.1039/d2dt02079c

**Published:** 2022-08-31

**Authors:** Alexandra Sink, Samya Banerjee, Juliusz A. Wolny, Cinzia Imberti, Edward C. Lant, Marc Walker, Volker Schünemann, Peter J. Sadler

**Affiliations:** Department of Chemistry, University of Warwick Gibbet Hill Road Coventry CV4 7AL UK P.J.Sadler@warwick.ac.uk; Department of Chemistry, Indian Institute of Technology (BHU) Varanasi UP-221005 India samya.chy@itbhu.ac.in; Department of Physics, Technische Universität Kaiserslautern Erwin-Schrödinger-Straße 46 67663 Kaiserslautern Germany wolny@rhrk.uni-kl.de; Department of Physics, University of Warwick Gibbet Hill Road Coventry CV4 7AL UK

## Abstract

We have studied activation of the methyl C–H bonds in the cyclopentadienyl ligands of half-sandwich Rh(iii) complexes [*η*^5^-Cp^X^Rh(N,N′)Cl]^+^ by observing the dependence of sequential H/D exchange on variations in Cp^X^ = Cp* (complexes 1 and 2), Me_4_PhCp (Cp^XPh^, 3) or Me_4_PhPhCp (Cp^XPhPh^, 4), and chelated ligand *N*,*N*′ (bpy, 1; phen, 2–4). H/D exchange was fastest in d_4_-MeOD (*t*_1/2_ = 10 min, 37 °C, complex 1), no H/D exchange was observed in DMSO/D_2_O, and d_4_-MeOD enhanced the rate in CD_3_CN. The proposed Rh(i)–fulvene intermediate was trapped by [4 + 2] Diels–Alder reactions with conjugated dienes and characterized. The Rh(i) oxidation state was confirmed by X-ray photoelectron spectroscopy (XPS). Influence of solvent on the mechanisms of activation and Diels–Alder adduct formation was modelled using DFT calculations with the CAM-B3LYP functional and CEP-31 g basis set, and influence on the reaction profile of the dimiine ligand and phenyl substituent using the larger qzvp basis set. The Rh(iii)–OH intemediate is stabilised by H-bonding with methanol and a Cp* CH_3_ hydrogen. The Rh(i)(Me_4_fulvene) species, stabilised by interaction of methanol with a coordinated water, again by two H-bonds H_2_O–HOMe (1.49 Å) and fulvene CH_2_ (1.94 Å), arises from synchronous transfer of the methanol OH proton to a Rh(iii)–OH ligand and Cp* methyl hydrogen to the methanol oxygen. Additionally, the observed trend in catalytic activity for complexes 1–4 was reproduced by DFT calculations. These complexes form a novel class of catalytic molecular motors with a tunable rate of operation that can be stalled in a given state. They provide a basis for elucidation of the effects of ligand design on the contributions of electronic, rotational and vibrational energies to each step in the reaction pathway at the atomic level, consideration of which will enhance the design principles for the next generation of molecular machines.

## Abstract 2

The roles of hydrolysis, concerted proton transfer, and methanol in ring methyl C–H activation *via* a Rh(i)–fulvene intermediate are elucidated.

## Graphical abstract

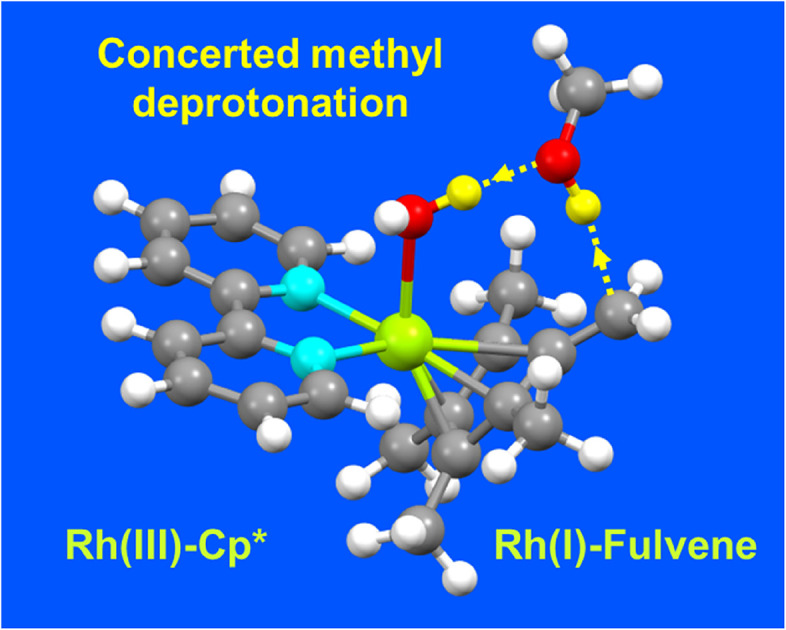

## Introduction

**Scheme 1:**
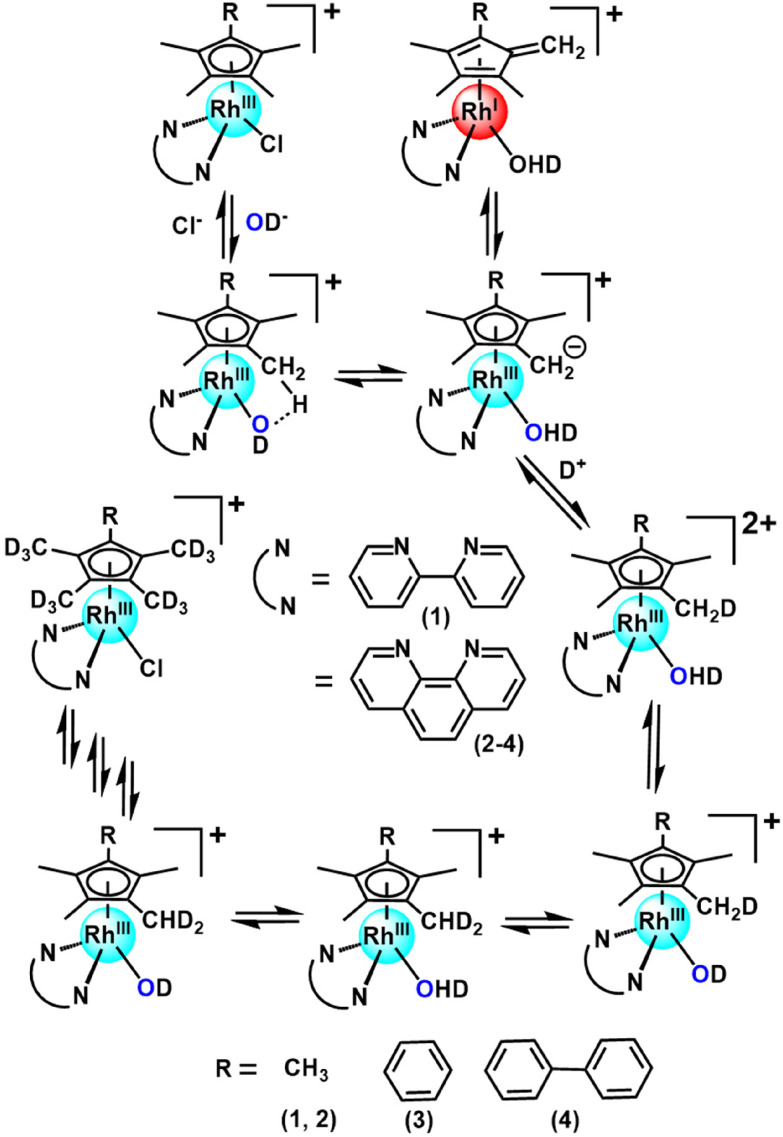
Proposed mechanism for the activation of ring methyl C–H bonds in this class of Rh(iii) cyclopentadienyl complexes containing methyl, phenyl or biphenyl Cp^X^ substituents and bipyridyl or phenanthroline *N*,*N*-chelated ligands.

Organometallic Rh(iii) half-sandwich complexes with the general formula [(Cp^X^)Rh(N/C,N′)X] where X = halide, Cp^X^ = pentamethylcyclopentadienyl (Cp*), tetramethyl(phenyl)cyclopentadienyl (Cp^xPh^) or tetramethyl(biphenyl)cyclopentadienyl (Cp^xPhPh^) are of interest as potential catalysts and anticancer and antimicrobial agents.^1–10^ In general, methyl Cp^X^ ring substituents in metal complexes are difficult to deprotonate. However, this can be achieved when the resulting –CH_2_^−^ fragment is stabilized, as in the case of pentamethylcyclopentadienyl (Cp*) ligands in some half-sandwich Rh(iii) complexes.^11–13^ We discovered that this type of complex can readily undergo ring methyl deprotonation and H/D exchange under mild conditions of physiological relevance (aqueous media, body temperature).^14^ Curiously, the presence of methanol appeared to enhance the rate of H/D exchange. We were able to trap a Rh(i)–fulvene intermediate in the proposed reaction mechanism by [4 + 2] cycloaddition reactions with the conjugated dienes (Scheme 1). Analogous Ir(iii) complexes do not undergo similar H/D exchange.^14^

**Fig. 1:**
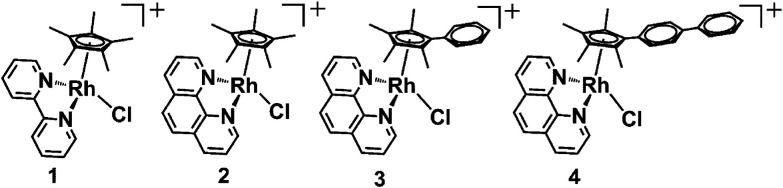
The half-sandwich organo-rhodium(iii) complexes studied in this work. All the complexes were isolated as PF_6_^−^ salts.

Recently, we used inelastic neutron scattering to record the vibrational spectra of [Cp*Rh(bipy)Cl]^+^ (complex 1, Fig. 1) and its deuterated D_15_-Cp* analogue **D**_**15**_**-1**.^15^ The vibrations were fully assigned using Density Functional Theory (DFT) phonon calculations.^15^ The vibrational analysis revealed that the Cp* ring behaves like a moving carousel, and brings each methyl proton close to the Rh–OH/D centre where proton abstraction occurs (Scheme 1). The DFT modelling revealed changes in vibrations along the whole reaction path, involving a Rh(i)–fulvene intermediate, Remarkably, vibronic energy contributions are large across the entire transition, amounting to over a 400-fold increase in the proton transfer rate. Rh(iii) behaves as a catalytic centre, sequentially deprotonating each of the Cp* methyl hydrogens, promoting their deuteration in the presence of a source of deuterons.

Related catalytic metal centres exist in natural metalloenzymes. For example, the Zn(ii) enzyme carbonic anhydrase is a highly efficient catalyst for the hydration of CO_2_, *via* a mechanism involving deprotonation of Zn–OH_2_ and Zn–OH attack on the carbon of CO_2_.^16^ The lowering of the p*K*_a_ for H_2_O is dependent not only on binding to Zn(ii), but also interactions with the nearby amino acid side chains, as well as the dynamic behaviour of water molecules.^16,17^ However, the contributions of individual atoms and in particular their vibrational motions, are currently beyond the scope of computational calculations, hence model catalytic systems such as that studied here are important for elucidation of the roles of individual atoms along catalytic pathways.

It is stimulating too to consider the possible parallels between these Rh(iii) coordination complexes and molecular machines. It has been noted that the fundamental issues associated with the dynamics of molecular systems have not been sufficiently addressed to allow the design of materials that produce work efficiently (molecular machines).^18^ It is interesting therefore to consider whether these Rh(iii) complexes exhibit any of the features of molecular machines: can they be switched on and off, or their work-rate modulated, and if so how? Can they be braked reversibly whilst in motion? And how do modifications to the ligands change their efficiency?

These considerations have led us to this study of the kinetics of methyl deuteration of Rh(iii) complexes [(*η*^5^-Cp^X^)Rh(N,N′)Cl]^+^, in which the cyclopentadienyl ring contains either 5 methyl substituents, or 4 methyls and a phenyl or biphenyl substituent (Cp^X^ = Cp*, Cp^xPh^ or Cp^xPhPh^), together with a chelated diimine, N,N′ = bipyridyl or phenanthroline (1–4, Fig. 1). We have studied the effects of the solvent and temperature on the rate of deuteration, as well as Diels–Alder [4 + 2] cycloaddition reactions as potential brakes for sequential ring methyl activation, with a range of conjugated aromatic and non-aromatic dienes. Importantly, we used X-ray photoelectron spectroscopy (XPS) to confirm the trapping of Rh(i) in the proposed Diels–Alder adducts. These experimental studies are complemented by detailed DFT calculations to elucidate the kinetics and mechanism of the reactions, including the influence of phenyl substituents on Cp^X^ and the role of the *N*,*N*′-chelated ligand (bipyridyl, phenanthroline). An interesting role for methanol in the proton transfer process emerges.

## Results

Complexes 1–4 (Fig. 1) were synthesized by reaction of the corresponding bidentate chelating ligand (2–3 mol equiv.) with the appropriate dimer, [(Cp^X^)RhCl_2_]_2_, following our reported procedure.^7,14^ They were characterized by ^1^H NMR and ESI-MS analysis (see the ESI†). Under very mild conditions (ambient temperature), the methyl-groups of the Cp^X^-ligands of the complexes can undergo activation and sequential deuteration (Scheme 1). First we studied the dependence of the kinetics of H/D exchange on the Cp^X^ and *N*,*N*-chelated ligands in complexes 1–4 in d_4_-MeOD/D_2_O (3 : 2 v/v), and then the dependence on the solvent, concentration of the complex, and temperature.

### Kinetics of deuteration

**Fig. 2:**
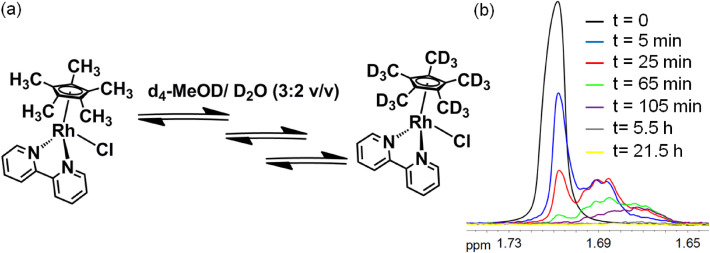
(a) Deuteration of complex 1. (b) Time-dependent CH_3_ deuteration of Cp*-followed by ^1^H NMR at 37 °C in d_4_ MeOD/D_2_O (3 : 2). The progressive shift to high field from CH_3_ to CH_2_D to CHD_2_ arises from isotope shifts (*ca.* 0.02 ppm per D) and the appearance of new multiplets from ^1^H-D coupling (^2^*J ca.* 2–2.4 Hz).

We followed the kinetics of deuteration of the complexes (0.5 mM) in d_4_-MeOD/D_2_O (3 : 2 v/v) at 37 °C by ^1^H-NMR spectroscopy. The singlet peak for 15 protons of the 5 Cp*–CH_3_ groups for complex 1 at 1.72 ppm decreased in intensity over a period of a few hours, accompanied by asymmetric splitting and broadening of the peak. The time dependence of the methyl ^1^H-NMR peaks is shown in Fig. 2, and is representative of the kinetic studies on complexes 1–4. The peak shifts as deuteration progresses due to a D-isotope effect. Deuterium coupling is also evident, giving a 1 : 1 : 1 triplet for a CH_2_D group (nuclear spin of D, I = 1, ^2^J(^1^H–D) coupling), and a 1 : 2 : 3 : 2 : 1 quintet for a CHD_2_ group.

**Fig. 3:**
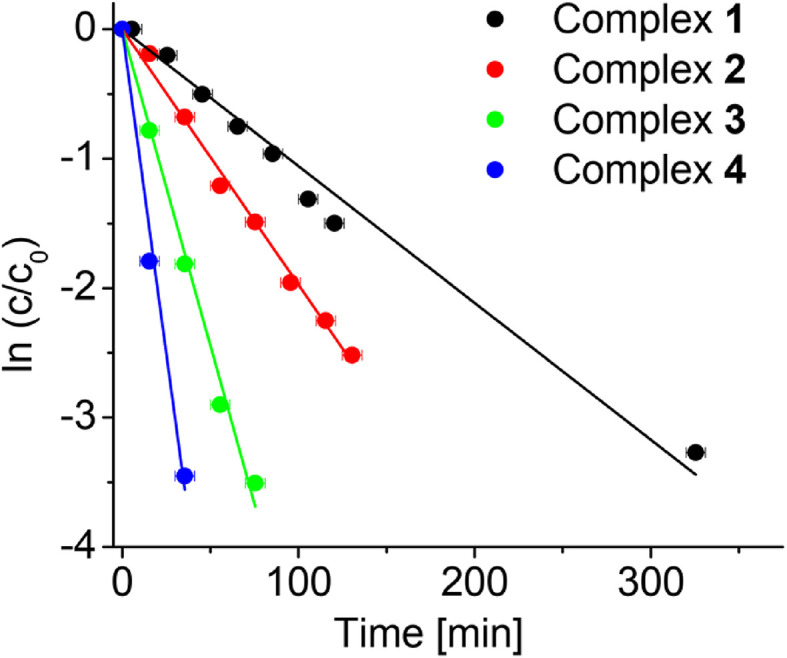
Extent of deuteration of complexes 1–4 (0.5 mM) with time in d_4_-MeOD/D_2_O (3 : 2 v/v) at 37 °C determined by ^1^H NMR spectroscopy. The lines represent first-order kinetic fits to the data with the rate constants shown in Table 1. *c*_0_ and *c* are the initial concentration of 1 and concentration of 1 at a particular time, respectively.

The rate of deuteration was highly dependent on the *N*,*N*′-chelating ligand as well as the substituent on the Cp^X^-ligand. The time dependence of the deuteration of complexes 1–4, with deuterium in large excess, follows pseudo 1^st^ order kinetics (Fig. 3). The rate of deuteration of the 1,10-phen complex 2 was *ca.* 2× times faster than that of the bipy complex 1 (Table 1). On changing a Cp^X^ substituent from CH_3_ (complex 2) to Ph (complex 3) or PhPh (complex 4), the rate of deuteration decreases by *ca.* 2.5× and 5×, respectively (Table 1).

**Table 1:** Rate constants and half-lives (*t*_1/2_) for deuteration of Rh(iii) complexes 1–4 in d_4_-MeOD/D_2_O (3 : 2 v/v) at 37 °C

Complex	*k* (min^−1^)	*t*_1/2_ (min)
1	0.011 ± 0.002	63.3
2	0.020 ± 0.002	35.1
3	0.049 ± 0.003	14.2
4	0.100 ± 0.005	6.91

#### Effect of solvent

The rate of deuteration of complex 1 (1 mM) at 37 °C in the solvent mixtures d_6_-DMSO/D_2_O (9 : 1, 3 : 2, 1 : 19 v/v), d_6_-acetone/D_2_O (9 : 1, 3 : 2, 1 : 19 v/v), d_3_-MeCN, d_4_-MeOD, d_3_-MeCN/d_4_-MeOD (9 : 1, 3 : 2, 2 : 3 v/v), and d_4_-MeOD/D_2_O (9 : 1, 3 : 2, 1 : 19 v/v) was determined by ^1^H NMR spectroscopy.

**Fig. 4:**
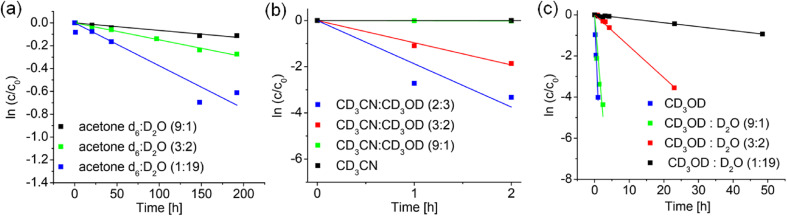
Variation of the rate of deuteration of complex 1 at 37 °C with solvent composition, in (a) d_6_-acetone/D_2_O, (b) d_3_-MeCN/D_2_O and (c) d_4_-MeOD/D_2_O, as determined by integration of ^1^H NMR peaks.

It can be seen from Table 2 that no deuteration was observed in any of the d_6_-DMSO/D_2_O mixtures, or in d_3_-MeCN (as expected, since no labile D available), although in the latter case, addition of d_4_-MeOD (9 : 1, 3 : 2, 2 : 3 v/v) induced deuteration, being quite rapid at 60% d_4_-MeOD/40% d_3_-MeCN (half-life 15 min). Deuteration was fastest in d_4_-MeOD (half-life 8 min), but was almost half as fast with 10% D_2_O present, and very slow in the presence of 95% D_2_O (half-life of 36 h, Table 2. Fig. 4a–c). Fig. 4a–c indicate that a protic solvent is essential for the deuteration and that the rate of deuteration increases with the proportion of protic solvent. However, the slowing down on addition of D_2_O to d_4_-MeOD, appears to indicate that methanol plays a special role (Fig. 4c).

**Table 2:** Rate constants and half-lives for deuteration of complex 1 in various solvents at 37 °C

Solvent	*k* (h^−1^)	*t*_1/2_
**d**_**6**_ **-DMSO**/D_2_O (9 : 1 v/v)	No deuteration	—
d_6_-DMSO/D_2_O (3 : 2 v/v)	No deuteration	—
d_6_-DMSO/D_2_O (1 : 19 v/v)	No deuteration	—
**d**_**6**_ **-Acetone**/D_2_O (9 : 1 v/v)	(6.6 ± 0.3) × 10^−4^	43.9 d
d_6_-Acetone/D_2_O (3 : 2 v/v)	(14.8 ± 0.7) × 10^−4^	19.5 d
d_6_-Acetone/D_2_O (1 : 19 v/v)	(37.5 ± 1.9) × 10^−4^	7.7 d
**d**_**3**_ **-MeCN**	No deuteration	—
d_3_-MeCN/d_4_-MeOD (9 : 1 v/v)	(8.6 ± 0.4) × 10^−3^	3.36 d
d_3_-MeCN/d_4_-MeOD **(3 : 2**v/v**)**	0.96 ± 0.05	43.3 min
d_3_-MeCN/d_4_-MeOD (2 : 3 v/v)	1.9 ± 0.1	21.8 min
**d**_**4**_ **-MeOD**	4.2 ± 0.2	10.0 min
d_4_-MeOD/D_2_O (9 : 1 v/v)	2.0 ± 0.1	20.8 min
d_4_-MeOD/D_2_O **(3 : 2**v/v**)**	0.15 ± 0.01	4.6 h
d_4_-MeOD/D_2_O (1 : 19 v/v)	0.02 ± 0.001	34.6 h

#### Dependence on complex concentration

**Fig. 5:**
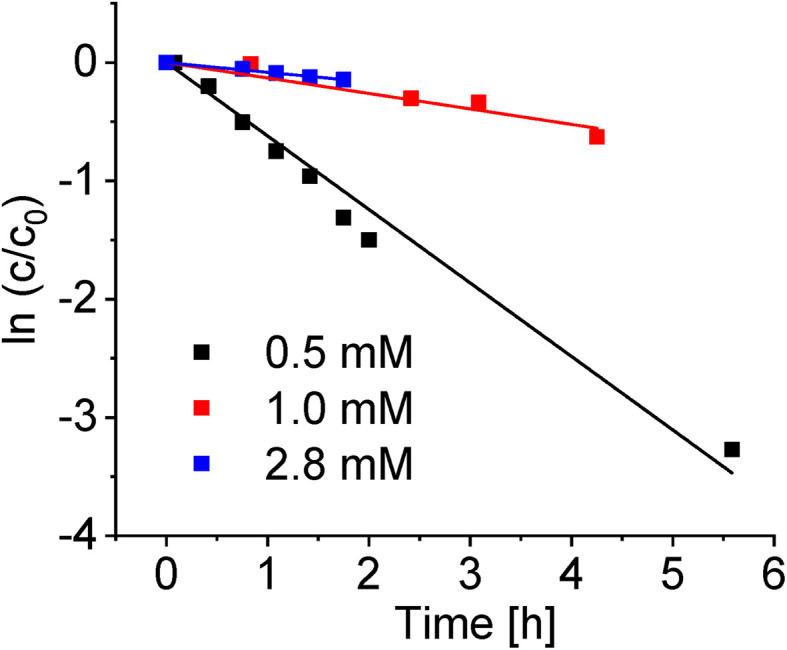
Variation in the rate of deuteration of complex 1 with concentration in d_4_-MeOD/D_2_O (3 : 2 v/v) at 37 °C. The lines represent the best fits to first order kinetics with the rate constants in Table 5.

Deuteration of complex 1 at concentrations of 0.5 mM, 1.0 mM and 2.8 mM in d_4_-MeOD/D_2_O (3 : 2 v/v) solutions at 37 °C was followed by ^1^H-NMR spectroscopy over time (Fig. 5). There was a significant decrease in the rate of deuteration with increase in the concentration of 1, by factors of *ca.* 5 and *ca.* 8 at concentrations of 1.0 mM and a 2.8 mM, respectively, compared to 0.5 mM, Fig. 5 and Table 3.

**Table 3:** Rate constants and half-lives for deuteration for complex 1 at various concentrations in d_4_-MeOD/D_2_O (3 : 2 v/v) at 37 °C

Concentration of 1	*k* (h^−1^)	*t*_1/2_ (h)
0.5 mM	0.62 ± 0.03	1.11
1.0 mM	0.13 ± 0.01	5.33
2.8 mM	0.08 ± 0.01	8.66

This increase in the rate of deuteration with lowering of the concentration of complex 1 is consistent with the suppression of solvolysis at higher concentration^14^ (2.8 mM) and relatively lower amount of the active aqua complex present compared to the more dilute solutions.

#### Temperature dependence

**Fig. 6:**
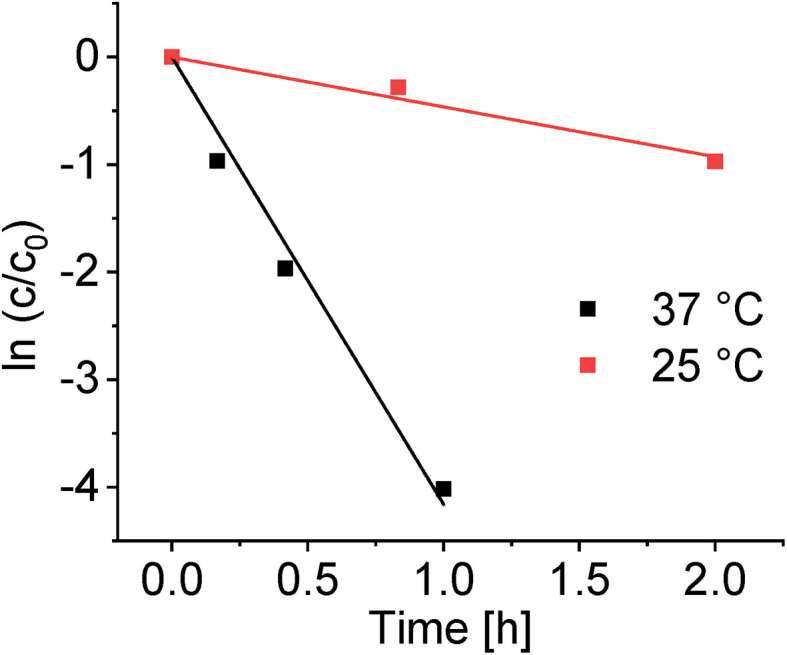
Dependence on time of the deuteration of complex 1 (1.0 mM) in d_4_-MeOD at 37 °C and ambient temperature (*ca.* 25 °C), as determined by integration of the Cp* ^1^H NMR peak.

Deuteration for a 1.0 mM solution of complex 1 in d_4_-MeOD was studied at ambient temperature (*ca.* 25 °C) and at 37 °C (Fig. 6 and Table 4). At 37 °C the rate of deuteration was *ca.* 9 × higher than at ambient temperature. In accordance with the Arrhenius equation (ln *k* = −*E*_a_/RT + ln *A*, where *E*_a_ is the activation energy), a 10 °C rise in temperature would be expected to double a reaction rate. The observed additional increase in rate can be ascribed to increased chloride dissociation at the higher temperature and increased concentration of the active aqua species.

**Table 4:** Temperature dependence of rate constants and half-lives for deuteration of complex 1 in d_4_-MeOD

Temperature	*k* (h^−1^)	*t*_1/2_ (h)
25 °C	0.46 ± 0.02	1.5
37 °C	4.2 ± 0.21	0.17

### Diels–Alder reactions

We have shown previously that deuteration proceeds *via* an intermediate that can be trapped by Diels–Alder cycloaddition reactions with conjugated dienes such as isoprene.^14^ In this work, we have studied such [4 + 2] Diels–Alder reactions with a range of aromatic (3,6-di-2-pyridyl-1,2,4,5-tetrazine and 1,3-diphenylisobenzofuran) and non-aromatic (isoprene, α-terpinene, sorbic acid, ethyl sorbate and (9Z,11E) conjugated linoleic acid) dienes, and also sought evidence for the intermediate being a Rh(i)–fulvene complex.

#### HRMS and X-ray photoelectron spectroscopy of Diels–Alder adducts

**Scheme 2:**
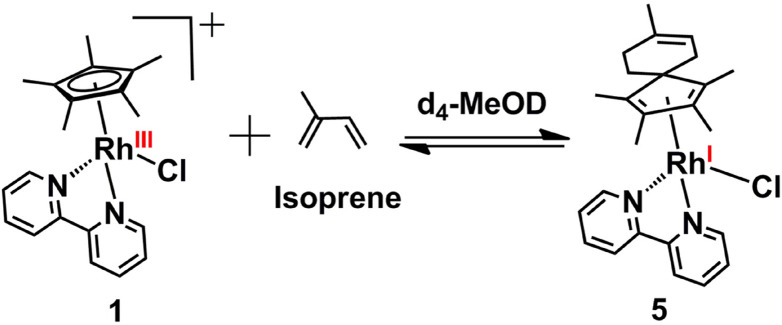
Diels–Alder reaction of Rh(iii) complex 1 with isoprene in d_4_-MeOD/D_2_O at 37 °C to generate Rh(i) complex 5.

The Diels–Alder adduct (complex 5), trapped from reactions of complex 1 with isoprene at ambient temperature in d_4_-MeOD (Scheme 2) was characterised in the crude reaction mixture by mass spectrometry (Fig. S1–S3, ESI†). It was difficult to isolate and purify this adduct, which was unstable and readily underwent dissociation/decomposition. Moreover, the yield (as determined by NMR analysis of the crude reaction mixture) of 5 was very low in repeated attempts. In the mass spectral analysis, the peak at *m*/*z* = 497.1225 is compatible with a Rh^I^ complex (5 + H)^+^ with the formula C_25_H_30_ClN_2_Rh (calc. *m*/*z* = 497.1231), which we reported previously.^14^ Mass spectral analysis also suggested that the Rh–Cl bond in 5 is weak, with the peak at 461.1496 corresponding to the loss of chloride [5-Cl]^+^ observable (calc. *m*/*z* = 461.1459; Fig. S1, ESI†). This is consistent with literature reports of a very long Rh^I^–Cl bond (*ca.* 2.55 Å) in a Rh^I^–(Cp*H) complex.^19^ Formation of adduct 5 was also confirmed by LC-MS analysis of the reaction mixture (Fig. S2, ESI†), which showed a reverse-phase HPLC peak at *ca*. 27.4 min with an *m*/*z* of 461.08, again assignable to [5-Cl]^+^.

**Fig. 7:**
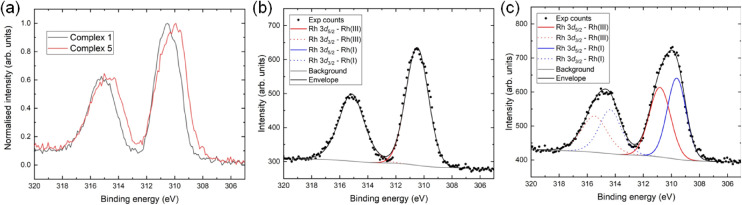
(a) Normalised Rh 3d XP spectra from complexes 1 and 5, showing a downward shift and broadening of the envelope upon reduction. (b) The Rh 3d region acquired from complex 1, shows the presence of only Rh(iii). (c) The Rh 3d region acquired from complex 5, illustrates the formation of Rh(i).

Next we used X-ray photoelectron spectroscopy to establish the presence of Rh(i) in the Diels–Alder adduct 5, and hence a Rh(i)-fulvene intermediate in the deuteration cycle (Scheme 1). The reduction of the initial Rh(iii) to Rh(i), is evident from comparison of the 3d_5/2_ electron binding energy in the X-ray photoelectron spectrum of complex 1 with that in the isoprene adduct 5. Fig. 7a shows the normalised Rh 3d regions acquired by XPS from complexes 1 and 5, showing a downward shift in the envelope of *ca.* 0.6 eV and an increase in the linewidth as a result of the reduction from Rh(iii) to Rh(i). Detailed fitting of the spectra revealed a single pair of components present for complex 1, Fig. 7b, with the Rh 3d_5/2_ peak at 310.5 eV being consistent with reported Rh(iii) compounds.^20^ Importantly, inclusion of a second pair of components, assigned to Rh(i), was necessary to replicate accurately the Rh 3d spectrum acquired from complex 5, Fig. 7c. The separation of the Rh(iii) and Rh(i) components is 1.20 eV, in line with previous reports.^21^ These results clearly demonstrate that the initial Rh(iii) is reduced to Rh(i) in the Diels–Alder adduct.

**Scheme 3:**
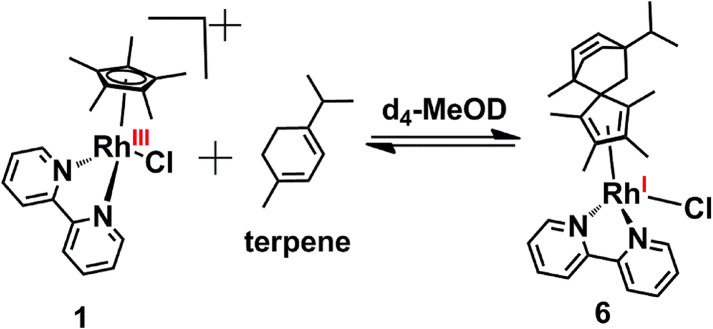
Diels–Alder reaction of complex 1 with α-terpinene in d_4_-MeOD/D_2_O at 37 °C to form complex 6.

Diels–Alder adduct formation for complex 1 was investigated for various other dienes. Complex 1 formed a Diels–Alder adduct with α-terpinene, complex 6 (Scheme 3, which was characterized by HR-MS (Fig. S3, ESI†). The peak at *m*/*z* = 529.2080 corresponds to [6-Cl]^+^ with the formulation C_30_H_38_N_2_Rh (calculated *m*/*z* = 529.2085).

XPS studies of the crude reaction mixture indicated the co-existence of the Rh(iii) and Rh(i) complexes in the reaction mixture *via* a slight broadening on the low binding energy side of the Rh 3d spectrum acquired from complex 6 compared to complex 1 (Fig. S4, ESI†). A more detailed analysis (Fig. S5, ESI†) showed that the binding energy difference between the Rh(iii) and Rh(i) complexes was 1.26 eV, consistent with the shift observed above. The ratio of Rh(i):Rh(iii) was 0.14 : 1 indicating that *ca.* 12% of Rh(i)-complex was present in the reaction mixture.

**Scheme 4:**
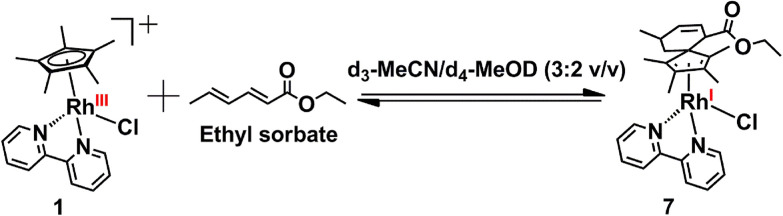
Diels–Alder reaction of complex 1 with ethyl sorbate in d_3_-MeCN/d_4_-MeOD (3 : 2 v/v) at 37 °C to form complex 7.

Complex 1 did not form a Diels–Alder adduct with sorbic acid, as was evident from the mass spectral analysis of the reaction mixture. However, complex 1 did form a Diels–Alder adduct with the ethyl ester of sorbic acid efficiently (Scheme 4). Reaction between complex 1 with ethyl sorbate in d_3_-MeCN/d_4_-MeOD (3 : 2 v/v) at 37 °C gave complex 7, which was characterized in the reaction mixture by mass spectrometry (Fig. S6 and S7, ESI†). The peak at *m*/*z* = 533.1765 corresponds to [7-Cl]^+^ with the formulation C_28_H_34_N_2_O_2_Rh (calculated *m*/*z* = 533.1675).

**Fig. 8:**
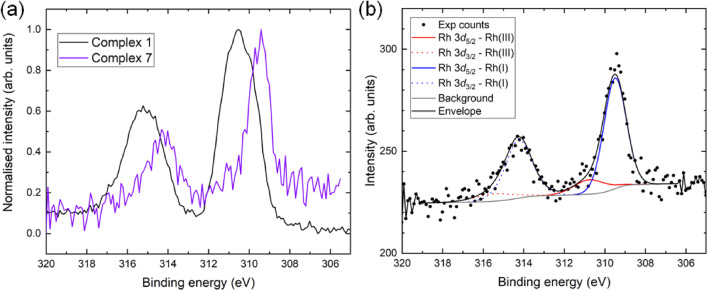
(a) Normalised Rh 3d XP spectra from complex 1 and its Diels–Alder adduct with ethyl sorbate, complex 7, showing a downward shift of the envelope upon reduction. (b) The Rh 3d region acquired from complex 7, showing that Rh(i) is the predominant species in the reaction mixture.

Comparison between the oxidation states of Rh in complex 1 and complex 7 was made by XPS for the crude reaction mixtures, with a noticeable downward shift of almost the entire Rh 3d signal achieved during adduct formation (Fig. 8a), clearly indicating that a significant proportion of the Rh(iii) is reduced to Rh(i). A more detailed analysis (Fig. 8b) showed that the difference in binding energy of Rh(iii) (complex 1) to Rh(i) (complex 7) is 1.27 eV, again consistent with the reactions above.

**Scheme 5:**
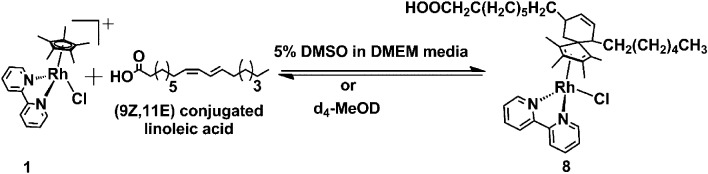
Diels–Alder reaction of Rh(iii) complex 1 with conjugated (9*Z*,11*E*)-linoleic acid to form Rh(i) complex 8.

Complex 1 also formed a cyclo-addition adduct with the conjugated diene (9*Z*,11*E*)-linoleic acid (complex 8, Scheme 5). Complex 8 was well characterized by HRMS, LC-MS (Fig. S8 and S9, ESI†). The HRMS data indicated the formation of complex 8 with the peak at *m*/*z* = 673.3229 assignable to C_38_H_54_N_2_O_2_Rh, the [8-Cl]^+^ (calculated *m*/*z* = 673.3240; Fig. S8, ESI†). Formation of complex 8 was also evident from the LC-MS analysis, giving rise to a reverse-phase HPLC peak at *ca*. 9.0 min with an *m*/*z* of 691.21 assignable to [8-Cl + H_2_O]^+^. From the XPS studies (Fig. S10 and S11, ESI†), *ca.* 33% formation of Rh(i) species was evident. We reported earlier that complex 8 undergoes dissociation on dilution,^14^ a feature of all the adducts studied here.

**Fig. 9:**
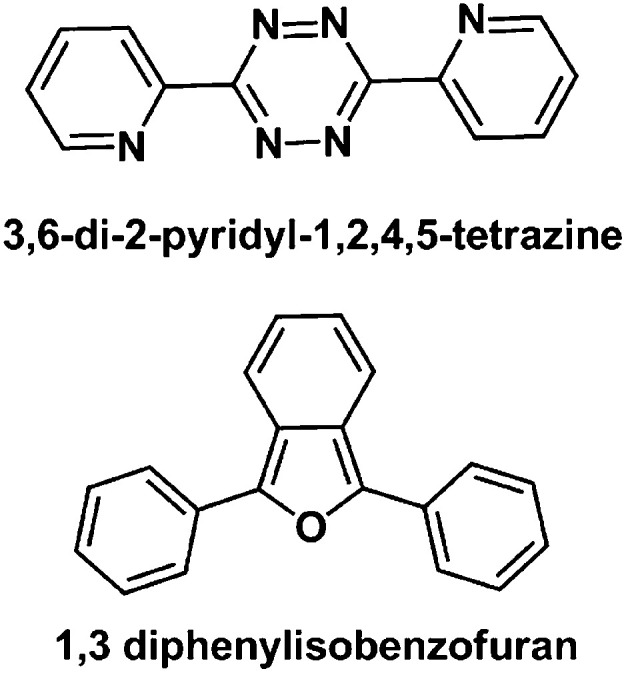
Structures of aromatic dienes studied in attempts to form [4 + 2] cyclo-addition adducts with complex 1.

An attempt to study the [4 + 2] cyclo-addition reaction between complex 1 (1 mol eqiv.) and the aromatic diene 3,6-di-2-pyridyl-1,2,4,5-tetrazine (10 mol eqiv., Fig. 9) in d_4_-MeOD/D_2_O (3 : 2 v/v) was not successful, as was evident from the mass spectral analysis. Complex 1 also did not react to form a [4 + 2] cyclo-addition adduct with another aromatic diene *viz.*, 1,3-diphenylisobenzofuran (Fig. 9) under identical reaction conditions.

### DFT modelling

Possible interactions between the species involved in the deuteration process and the influence of the solvent were modelled using DFT calculations. The calculations were performed with Gaussian 16^22^ using the CAM-B3LYP functional^23^ and CEP-31 g basis set^24^ with modelling of methanol as solvent. In a further step, the influence on the reaction profile of the dimiine ligand and a phenyl substituent on Cp* ligand was investigated with the larger data basis qzvp,^25^ again with modelling of methanol as solvent. Firstly, we calculated the relative energies of the [Rh(N,N′)(Cp*-R)OH]^+^·MeOH and [Rh(N,N′)(Me_4_R-fulvene)OH_2_]^+^·MeOH species, as well as that of the square-planar [Rh(N,N′)(Me_4_R-fulvene)·OH_2_]^+^·MeOH isomers. Then we also modelled the aquation of the [Rh(N,N′)(Cp*-R)Cl]^+^ precursors with the same theory. The details are given in the ESI.†

Within this study, the electronic energies of six species with bipyridine as diimine ligand were calculated. First for the molecular assemblies in vacuum, and then with modelling of methanol as solvent within the IEFPCM method:^25^

(a) [Rh(bipy)(Cp*)(OH)]^+^ with a non-interacting molecule of MeOH,

(b) [Rh(bipy)(Cp*)(OH)]^+^·MeOH, with a MeOH molecule interacting with the OH^−^ ligand,

(c) [Rh(bipy)(Cp*)(MeOH)]^2+^ + OH^−^; with a coordinated methanol and quasi-isolated hydroxide,

(d) [Rh(bipy)(Cp*)(OMe)]^+^ + H_2_O; with coordinated methoxide and quasi-isolated H_2_O,

(e) [Rh(bipy)(Me_4_fulv)(OH_2_)]^+^ + MeOH, the aqua complex of tetramethyl fulvene (Me_4_fulvene) and isolated methanol, and

(f) [Rh(bipy) (Me_4_fulv)OH_2_]^+^·MeOH; the aqua complex of Me_4_fulvene and an interacting methanol.

**Fig. 10:**
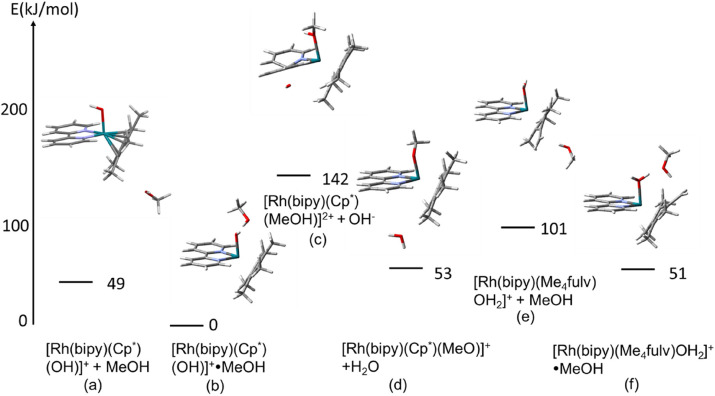
Calculated electronic energies (CAM-B3LYP/CEP-31g) (kJ mol^−1^) of various species involving the Cp* and Me_4_-fulvene adducts of complex 1 interacting with MeOH and MeO^−^, calculated with modelling of methanol as solvent. Note that the diamine ligand is shown always on the left of the complex, while the Cp*/Me_4_-fulvene is on the right. The weak contacts H**O**⋯**H**(O_MeOH_), **H**((OH)_MeOH_)⋯**C**((CH_2_=) _Cp_*) and **H**(water)⋯**O**(MeOH) are shown with dashed lines. Colour code: Rh – turquoise, N – blue, O – red, C – black, H – white/grey. The notation “(a)–(f)” corresponds to the species listed in the text above.

The results for modelling with methanol as solvent are shown in Fig. 10. The corresponding results for the modelling in vacuum are given in Fig. S12, in ESI.†

For both models, the most stable species is the Rh–OH^−^ complex with MeOH interacting with the hydroxyl group. The MeOH⋯OH distance of 1.59 Å indicates hydrogen bonding. Additionally, one of the hydrogens of the Cp* methyl group appears to interact with the MeOH oxygen, at a distance of 2.21 Å. These two interactions are responsible for the stabilisation of the species compared to that with the non-interacting methanol molecule, with energetic effects of 54 and 49 kJ mol^−1^ for the models in vacuum and with solvent, respectively.

Further, it seems that coordination of Rh(iii) to MeOH or MeO^−^ replacing the hydroxide (yielding a water molecule in the second case) is energetically unfavourable, leading to an increase in energy of 182 and 142 kJ mol^−1^ for the vacuum and solvent models, respectively, and 98 and 53 kJ mol^−1^ for the former and latter, respectively. At this point, however, it has to be noted that, contrary to the hydroxyl-coordinated structure (b) that is stabilised by the explicit H-bond, the structures with coordinated methanol include no (vacuum model) or unspecific interaction with solvent (solvent model). This is related to the fundamental problem that strong specific solvation of water or methanol cannot be taken into account by DFT calculations, while the PCM solvation model used here takes into account only non-specific solvation and not the hydrogen bonds, see ref. 26. On the other hand, the Me_4_fulv species also seems to be stabilised by the interaction of methanol with a coordinated water molecule, with an energetic effect of 74/50 kJ mol^−1^. Also in this case, two hydrogen bonds are predicted by DFT, H_2_O⋯HOMe (1.49 Å) and fulvene carbon⋯O(H)Me (1.94 Å). The DFT-optimised structures of these species are provided as pdb files in the ESI† ([Rh(bipy)(CpMe_5_)(MeO)]H_2_O.pdb; [Rh(bipy)(CpMe_5_)(MeOH)]OH.pdb; [Rh(bipy)(CpMe_5_)(MeOH)OH].pdb; [Rh(bipy)(CpMe_5_)OH]MeOH.pdb; [Rh(bipy)(Me_4_fulvene)(MeOH)OH_2_].pdb; [Rh(bipy)(Me_4_fulvene)OH_2_]MeOH.pdb). Apart from the case of [Rh(bipy)(Me_4_fulv)(OH_2_)]^+^·MeOH, the solvent effects seem to stabilise all complexes compared to [Rh(bipy)Cp*(OH)]^+^·MeOH. These results point towards the importance of the methanol molecule for stabilisation of the hydroxide- and aqua-coordinated Rh(iii) species, rather than to direct coordination of methanol or methoxide.

#### Modelling the kinetics of deuteration

Next, we attempted to model the kinetics of deuteration, involving hydrogen transfer from the Cp* methyl group with the formation of the Me_4_fulvene species. Again, the system with bipyridine as diimine ligand was chosen.

We studied a different mechanism for the proton transfer, by considering the species in which the methanol molecule interacts with the axial hydroxido or aqua ligand, as shown in Fig. 10. Accordingly, the QST calculations were performed for complex 1. The relative values obtained for both reactant and product, as well that of the transition state are given in Table 5. The comparison with the previously considered^23^ model of intramolecular proton transfer (with no methanol) molecule shows that both mechanisms could be operative. Similar to that mechanism, also a significant decrease of the activation barrier due to vibrational energy is observed for the formation of the fulvene mediated by a methanol molecule. The activation barrier is lowered to 17 and 15 kJ mol^−1^ for the forward and backward processes, respectively.

**Table 5:** Calculated electronic energies (CAM-B3LYP/CEP-31g) (in italics) and sum of electronic and vibrational energies of the transition state (shown in Fig. 12) for proton transfer between Rh–OH hydroxide and Cp^X^ methyl groups for complex 1, calculated for the models involving a methanol molecule compared to those obtained for the model assuming purely intramolecular (no methanol molecule involved).^15^

Mechanism	Axial ligand		
	Δ*E* (kJ mol^−1^) elec/elec + vib		
	OH	H_2_O	TS
Model with MeOH	0	51/49	79/62
Model with no MeOH	0	50/46	74/58

**Fig. 11:**
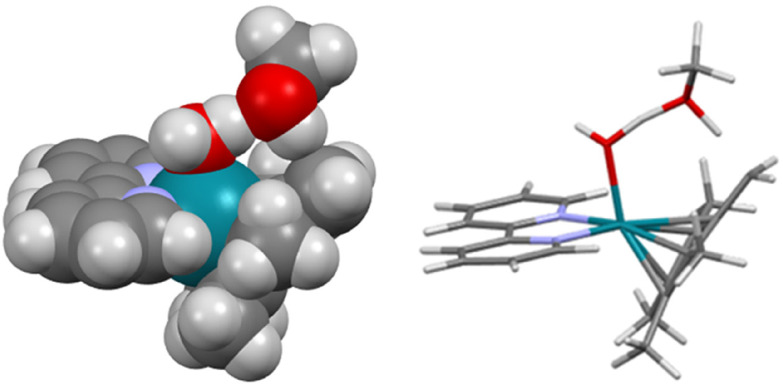
Calculated structure of the saddle point of the [Rh(bipy)Cp*(OH)]^+^·MeOH ↔ [Rh(bipy)(Me_4_-fulv)(OH_2_)]^+^·MeOH interconversion. Left: space-filling model. Right: stick model (to aid identification of atoms). The corresponding intermolecular distances are **H**(water)⋯**O**(MeOH) 1.26 Å, **H**((OH)_MeOH_)⋯**C**((CH_2_=)_Cp_*)1.39 Å. Colour code: Rh – turquoise, N – blue, O – red, C – black, H – white/grey.

This mechanism assumes that a fulvene species arises by synchronous transfer of the methanol OH hydrogen to the hydroxido ligand and the Cp* methyl hydrogen to the methanol oxygen (see the movies “imag_freq_model_vacuum.gif” and “imag_freq_solvent_model.gif” of the active vibration in the ESI†). The structure of the saddle point is shown in Fig. 11. It is stabilised by a chain of four weak O–H and C⋯H hydrogen bonds spanning between the coordinated oxygen and the active Cp* methyl group.

In order to account for the ligand dependence of the catalytic activity, we performed two types of calculations using the qzvp basis set together with the CAM-B3LYP functional. This approach is usually thought to give the better values of relative energies for different stages of the reaction under study. In the first step, we looked at the relative electronic energies of the hydroxido-species [Rh(N,N′)(Cp*-R)(OH)]^+^·MeOH and [Rh(bipy)(Me_4_-fulv)(OH_2_)]^+^·MeOH species as well as the pseudo square-planar [Rh(bipy)(Me_4_-fulv)]^+^·MeOH·H_2_O isomers. It is important to note that for the substituted Cp*-ligand, two conformations of the complex are possible, with the phenyl or biphenyl substituent located either on the side of *N*,*N*′-Rh plane with coordinated oxygen ligand, or on the opposite one, resulting in *E* and *Z* conformers (see ESI† for details and Fig. S13, ESI†). The relative energies of [Rh(N,N′)(Cp*-R)(OH)]^+^·MeOH [Rh(N,N′)(Me_4_-fulv)(OH_2_)]^+^·MeOH and square planar [Rh(N,N′)(Me_4_-fulv)]^+^·MeOH·H_2_O are collected in Table 6.

**Table 6:** Electronic energies (kJ mol^−1^) of [Rh(N,N′)(Cp*-R)(OH)]^+^·MeOH, [Rh(N,N′)(Me_4_-fulv)(OH_2_)]^+^·MeOH and square-planar [Rh(N,N′)(Me_4_-fulv)]^+^·MeOH·H_2_O for different ligand systems calculated with CAM-B3LYP/QZVP and methanol as solvent

N,N′, Cp*-ligand/conformation	Axial ligand/energies (kJ mol^−1^)		
	OH	H_2_O	Square-planar^a^
bipy, Cp*	0	47	44
phen, Cp*	0	45	43
phen, *E*-Cp*Ph	0	40	40
phen, *Z*-Cp*Ph	0	43	44
phen, *Z*-Cp*biph	0	40	40
phen, *E*-Cp*biph	0	42	40

a Square-planar coordination of Rh with only diimine and Cp*-fulvene ligands bound.

**Fig. 12:**
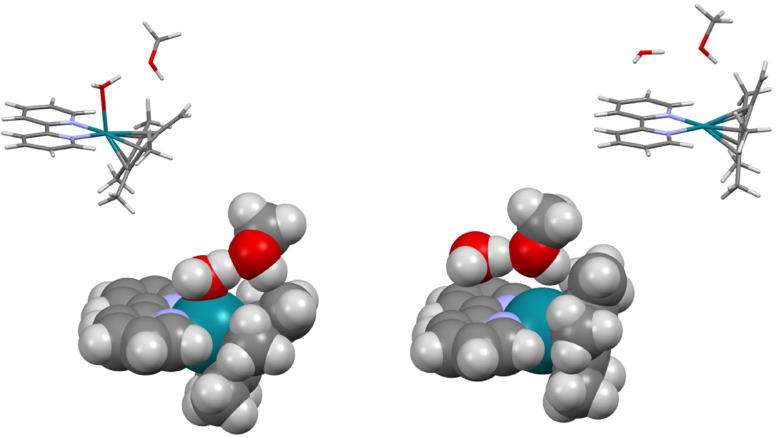
Comparison of the optimised (CAM-B3LYP/QZVP) structures of [Rh(bipy)(Me_4_-fulv)OH_2_]^+^·MeOH (left) and square-planar [Rh(bipy)(Me_4_-fulv)]^+^·MeOH·H_2_O (right). The insets on the top show the structures as stick models. Colour code: Rh – turquoise, N – blue, O – red, C – black, H – white.

Before discussing the trends in energy obtained that may be relevant to catalytic activity, it is interesting to look at the models of the square-planar species. The data collected in Table 6 reveal that their stability is similar to that of the corresponding aqua-coordinated [Rh(N,N′)(Me_4_-fulv)OH_2_]^+^·MeOH complexes. A comparison of the optimised structures of both water-coordinated and square-planar fulvene species is shown in Fig. 12.

For both systems, the fulvene ligand is stabilised by the methanol OH–C fulvene contact, being 2.094 and 2.330 Å for the aqua and square-planar species, respectively. The mutual orientation of the bipy and Cp* ligand is more close to perpendicular for the square-planar system with the N–Rh–*C*_fulvene_ angle being 137°, while that for the aqua-species is 124°. The most interesting difference, however is the *ca.* 0.04 Å shortening of the Rh–N bonds on going from aqua to square-planar species. The above effect is found for all modelled systems (see Table S1†) and may be indicative of the more pronounced Rh(i) character of the square-planar isomers.

The calculated relative electronic energies (CAM_B3LYP/QZVP) of the Cp* *vs.* fulvene (aqua and square-planar) reveal a gradual trend in the 1–4 series (Table 6), a *ca.* 10% decrease of the electronic energy of fulvene species *vs.* hydroxido complex between 1 and 4 in line with the observed increase in the catalytic activity. Thus the electronic factors, like the π-acceptor character of bipy and phen ligands, as well as the ability of the substituted Cp* ligands to stabilize the fulvene forms, bring about the increase of turnover in the 1–4 sequence. In order to obtain insight into other possible sources of this dependence, we examined the reactivity of the catalyst precursors [Rh(N,N′)(Cp*-R)Cl]^+^ towards forming the hydroxido-species [Rh(N,N′)(Cp*-R)(OH)]^+^. We modelled the process of the subsequent aquation of the precatalyst followed by the deprotonation of the coordinated water leading to formation of the hydroxido-species. The following reaction was therefore modelled: [Rh(N,N′)(Cp*-R)Cl]^+^ + 2H_2_O → [Rh(N,N′)(Cp*-R)(H_2_O)]^2+^ + H_2_O + Cl^−^ → [Rh(N,N′)(Cp*-R)(OH)]^+^ + H_3_O^+^ + Cl^−^. See the ESI† for details with the obtained electronic energies collected in Table S2.†

**Fig. 13:**
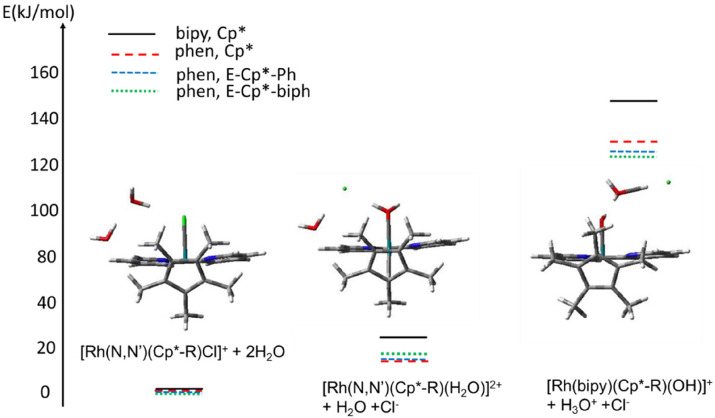
Estimated energy profile for the aquation/deprotonation reaction of the [Rh(N,N′)(Cp*-R)Cl]^+^ complexes calculated with CAM-B3LYP/QZVP and methanol as solvent for various ligand systems. The corresponding structures of [Rh(bipy)(Cp*-R)Cl]^+^ + 2H_2_O (left) _,_ [Rh(bipy)(Cp*-R)(H_2_O)]^2+^ + H_2_O + Cl^−^ (middle) and [Rh(bipy)(Cp*-R)(OH)]^+^ + H_3_O^+^ + Cl^−^ (right) systems are shown. Note that the *N*,*N*′-diimine ligand is shown in the background with its plane perpendicular to the figure plane. Colour code: Rh – turquoise, N – blue, Cl – green, O – red, C – black, H – white.

The results are collected in Table S2, ESI,† and shown schematically in Fig. 13. Due to the general problem of estimation of the explicit solvent interaction with DFT mentioned above and discussed in ref. 26, the absolute energies should be treated as estimates.

The results obtained imply that the nature of the *N*,*N*′-diimine ligands and the Cp*-substituent influence the ease of both aquation of the [Rh(N,N′)(Cp*-R)Cl]^+^ precursor and the deprotonation of the formed aqua [Rh(N,N′)(Cp*-R)(H_2_O)]^2+^ species to the hydroxide [Rh(N,N′)(Cp*-R)(OH)]^+^ complexes. Similar to the previously discussed influence of the ligands on the formation of the fulvene species, both the aquation and the dissociation of coordinated water become more facile on going from a bipyridine to a phenanthroline ligand. For the phen complexes, the substitution of one of the Cp* methyl groups with a phenyl or biphenyl group leads to a decrease in the relative electronic energies of the aqua and hydroxido complexes relative to the initial chlorido complex, in the sequence:

*E*_el_(Cp*-biphenyl) > *E*_el_(Cp*-phenyl) > *E*_el_(Cp*) for [Rh(phen)(Cp*-R)(H_2_O)]^2+^ and [Rh(phen)(Cp*-R)(OH)]^+^.

Thus, both DFT-predicted aquation of initial chlorido catalytic precursor leading to formation of the aqua and hydroxido species, and the ability of the chloride complex to convert to an aqua-fulvene species increase in the 1–4 sequence, in line with the observed catalytic activity.

## Discussion

Half-sandwich methyl-cyclopentadienyl-based Rh(iii) complexes of the type [Rh(N,N′)Cp^X^ Cl]^+^ possess the remarkable ability to undergo ring methyl C–H activation under mild conditions. Here we have gained new insight into the mechanism of such activation reactions from kinetic studies of the dependence on substituents on the Cp^X^ ring, the *N*,*N*-chelated ligand, the solvent, and the temperature, together with DFT modelling of the pathway. This also involved the trapping of Rh(i) fulvene intermediates by formation of Diels–Alder adducts with conjugated dienes, for which the rhodium oxidation state was characterised by XPS.

The *N*,*N*′-chelating ligand and also phenyl and biphenyl substituents on the Cp^X^-ligand, have a major influence on the rate of deuteration. The deuteration rate of the 1,10-phenthroline (phen) complex 2 is *ca.* 2× faster than that of the bipy complex 1 (Table 1). This correlates with the expected higher stabilization of the Rh(i)–fulvene intermediate by phen which is a stronger π-acceptor than bipy. Changing the Cp^X^ substituent on phenanthroline complexes from –CH_3_ (complex 2) to –Ph (complex 3), to –PhPh (complex 4), reduces the rate of deuteration by *ca.* 3× and 6× times (Table 1), respectively, which may be related not only to the removal of one methyl group, but also the increase in hydrophobicity, and steric effects resulting in a decrease in rate of rotation of the Cp^X^ ring.

The deuteration process is highly sensitive to the deuterated solvent used (Table 2). The presence of a protic solvent is essential for the deuteration, and the presence of d_4_-MeOD always increased the rate of deuteration. DFT calculations reveal the importance of methanol for stabilising, by H-bonding, not only for the hydroxido- and aqua Rh(iii) species, but also the Rh(i)(Me_4_fulvene) species, important intermediates in the deuteration process (Fig. 13). The rate of deuteration is also dependent on the concentration of the complex and temperature of the deuteration.

The DFT modelling implies that two possible factors are responsible for the observed experimental trend in turnover for the series of complexes 1–4. The stronger one is the influence of the diimine ligand/Cp* substituent on the aquation of the chloride-coordinated precursor influencing the energy needed to form the initial Rh–hydroxido/Cp* species. The somewhat weaker one is the dependence of the relative energy of the aqua- and square-planar fulvene species relative to the hydroxide/Cp^X^ species. Both factors operate in the same direction, leading to a higher catalytic efficiency for the phenanthroline complex with the increasing size of the Cp^X^-substituent.

The proposed fulvene intermediate was trapped by [4 + 2] cycloaddition reactions with a range of non-aromatic dienes such as isoprene, α-terpinene, ethyl sorbate and (9*Z*,11*E*) conjugated linoleic acid (Schemes 2–5). The extent of formation of the Rh(i) species with α-terpinene as the diene was much lower than that for isoprene, based on XPS data. This may be due to the increased steric crowding and structural rigidity around the conjugated double bond of α-terpinene. The observation that complex 1 did not form a Diels–Alder adduct with sorbic acid, but readily gave an adduct with ethyl sorbate, can be attributed to the presence of the electron-withdrawing carboxyl group in sorbic acid, which decreases the effective electron density over the conjugated double bonds. The carboxyl group in conjugated (9*Z*,11*E*)-linoleic acid may also play a role in reducing the extent of formation of adduct 8 (based on XPS) on reaction with complex 1, but in this case it is remote from the double bonds. XPS data confirmed the presence of Rh(i) in these fulvene intermediates.

Since it proved very difficult to isolate pure [4 + 2] cyclo-addition adducts of complex 1 with various dienes, we aimed to shift the equilibrium for the reaction completely towards adduct formation by liberation of dinitrogen from the [4 + 2] cyclo-addition reaction of complex 1 and 3,6-di-2-pyridyl-1,2, 4,5-tetrazine. However, this attempt was not successful, as complex 1 did not react with 3,6-di-2-pyridyl-1,2, 4,5-tetrazine. It seems likely that the endocyclic diene in this aromatic system has little tendency to behave as an isolated diene for the cyclo-addition reaction with the short lived exocyclic C=C of the Rh(i)-fulvene intermediate. A similar result with another aromatic diene (1,3-diphenylisobenzofuran) further supported this suggestion.

We consider now how the results obtained here contribute to the understanding of this class of complexes as molecular machines, in line with the standard definition as “molecules with controllable movements, which can perform a task when energy is added”.^27^ Firstly these half-sandwich Rh(iii) complexes are a unique type of catalytic molecular machine. They can be switched on by substitution of the chloride ligand by water which deprotonates and abstracts a proton from the Cp* ring. The resulting Rh(i) fulvene complex formed as an intermediate can then accept D^+^ from a donor solvent and undergo the back reaction to form a lower energy (H_14_D)-Cp* ligand. The rotational energy of Cp* allows it to move like a carousel, and in turn the next C–H of the Cp* ring becomes deuterated until all 15 H are replaced by D sequentially. At this point the system reaches a stationary state.

The simplicity of this machine lends itself to detailed investigations of the role in its efficiency at the atomic level not only of electronic effects, but also of rotational and vibrational energies. This has yet to be achieved for previously reported molecular machines.^28,29^ Our initial vibronic studies^15^ have revealed that vibrations are crucial for the reaction pathways, particularly, the conversion of local vibrations into translational motion for progression along the reaction coordinate, and for weakening bonds in the transition state, so lowering the vibrational energy.

The energetics for the various steps in the catalytic system under study are illustrated in Fig. S14.† The uphill part of the cycle leads to an increase of the electronic energy of the complex gained from the interaction with the solvent (with additional entropic effects) and heat. At the state of formation of the transition state between the hydroxide and fulvene species, the vibronic energy decreases. The deuteration due to interactions with the solvent again leads to a decrease of the vibrational energy of the complex. The decrease amounts to a total value of more than 100 kJ mol^−1^ in the fully deuterated 15D-steady state. Additionally, an increase of the vibrational entropy of more than 30 e.u (298.15 K) is estimated. Thus, the vibrational contribution seems to be both the driving force of the deuteration and decreases the free enthalpy of the transition state at least in one of the reaction stages.

## Conclusions

The activation of C–H bonds of Cp^X^–CH_3_ groups in half sandwich Rh(iii) complexes and their sequential deuteration under very mild conditions, raises interesting questions about the mechanism and the role of solvents in this process. The kinetics of such deuterations indicate that the auxiliary *N*,*N*′-donor chelating ligand has a significant role in controlling the rate of deuteration, as well as bulky substituents on the Cp^X^ ligand. Polar protic solvents in particular enhance the rate of deuteration and play a key role in this C–H activation. DFT calculations revealed the role of methanol in stabilising both the hydroxide/aqua-Rh(iii) as well as the Rh(i)-fulvene intermediates.

The DFT results obtained in this paper and in ref. 15 point towards the importance of the vibrational energy/entropy in providing the driving force for the deuteration reaction and lowering the activation energy for at least one reaction stage. The deuteration proceeds *via* a Rh(i)–fulvene intermediate which can form Diels–Alder [4 + 2] cycloaddition adducts with non-aromatic conjugated dienes, but not with the endocyclic conjugated double bonds of aromatic systems. Both steric and electronic factors control the reactivity of the conjugated diene. XPS analysis confirms the formation of the Rh(i)–fulvene [4 + 2] cycloaddition adduct intermediate, the first time that direct evidence for such an intermediate has been obtained.

This class of half-sandwich Rh(iii) cyclopentadienyl complexes is of interest for their anticancer properties and as catalysts for transfer hydrogenation reactions, Our studies suggest that facile tritium labelling of the methyl groups on bound Cp^X^ rings should be possible and could find use in radiotracer studies. These interesting reactions under the conditions of biological relevance, open up new opportunities for the design of therapeutic agents with new mechanisms of action and as well as new routes for synthesizing Diels–Alder adducts, which can be exploited in future work.

The system under study fulfils the criteria usually adopted for definition of a molecular machine. It is a molecular motor with a rotating Cp* ring. It seems that the combination of the interaction with solvent leading to aquation/solvation of the initial chloride precursor and the vibrational energy effects allow the machine to work. The work of the machine, *i.e.* exchange of all Cp* methyl protons, can be considered as exchange of the less elastic strings (*i.e.* the vibrations involving the Cp* methyl C–H bonds) with more elastic ones (the corresponding C–D vibrations). Considering all these factors, additional systematic changes of the substituents, both by experimental synthesis and DFT modelling, are likely to provide further insight into procedures which will optimise the design and increase the efficiency of molecular machines.

## Conflicts of interest

There are no conflicts to declare.

## Supplementary Material

DT-051-D2DT02079C-s001

DT-051-D2DT02079C-s002

DT-051-D2DT02079C-s003

DT-051-D2DT02079C-s004

DT-051-D2DT02079C-s005

DT-051-D2DT02079C-s006

DT-051-D2DT02079C-s007

DT-051-D2DT02079C-s008
