# Supplementary material for: Kinetics and mechanism of sequential ring methyl C–H activation in cyclopentadienyl rhodium(iii) complexes
Source: Dalton Trans. 2022 Aug 31;51(42):16070–81. doi: 10.1039/d2dt02079c (PMC9623609; doi:10.1039/d2dt02079c)
Supplement: DT-051-D2DT02079C-s001 [file DT-051-D2DT02079C-s001.pdf]

Electronic Supplementary Material (ESI) for Dalton Transactions.
This journal is © The Royal Society of Chemistry 2022

Electronic Supporting Information (ESI)

Kinetics and Mechanism of Sequential Ring Methyl C-H Activation in Cyclopentadienyl Rhodium(III) Complexes

Alexandra Sink,[§a] Samya Banerjee,[§*a,b] Juliusz A. Wolny,[§*c] Cinzia Imberti,[a] Edward C. Lant,[a] Marc Walker,[d] Volker Schünemann,[c] and Peter J. Sadler*[a]

[a] *Department of Chemistry, University of Warwick, Gibbet Hill Road, Coventry CV4 7AL, United Kingdom, P.J.Sadler@warwick.ac.uk*

[b] *Department of Chemistry, Indian Institute of Technology (BHU), Varanasi, UP-221005, India, samya.chy@itbhu.ac.in*

[c] *Department of Physics, Technische Universität Kaiserslautern, Erwin-Schrödinger-Straße 46, 67663 Kaiserslautern, Germany, wolny@rhrk.uni-kl.de*

[d] *Department of Physics, University of Warwick, Gibbet Hill Road, Coventry CV4 7AL, U. K.*

[§] *Equal contributions, joint first authors*

Contents

S.1 Materials and Methods

S.2 Synthesis and characterization of complexes **1-4**

S.3 Diels-Alder reactions

S.4. DFT calculations

Table 1: Calculated Rh-$N_{diimine}$ distances in $[Rh(N,N')(Cp^*-R)OH]^+$·MeOH, $[Rh(N,N')(Me_4\text{-}fulv)OH_2]^+$ ·MeOH and square planar $[Rh(N,N')(Me_4\text{-}fulv)]^+$ ·MeOH·H_2O systems.

Table S2. Calculated relative energies of the stages of the aquation/deprotonation of the $[Rh(N,N')(Cp^*-R)Cl]^+$ complexes in methanol.

Fig. S1 HRMS characterization of complex **5**.

Fig. S2 LC-MS characterization of complex **5**.

Fig. S3 HRMS characterization of complex **6**.

Fig. S4 Normalised Rh 3d XP spectra for complexes **1** and **6**.

Fig. S5 XPS Rh 3d region acquired from complex **6**, illustrating formation of Rh(I).

Fig. S6 HRMS characterization of the Diels-Alder adduct, complex **7**.

Fig. S7 LC-MS characterization of complex **7**.

Fig. S8 HRMS characterization of complex **8**.

Fig. S9 LC-MS characterization of complex **8**.

Fig. S10 Normalised Rh 3d XP spectra from complexes **1** and **8**.

Fig. S11 Rh 3d region acquired from complex **8**, illustrating the formation of Rh(I).

Fig. S12 Calculated electronic energies (CAM-B3LYP/CEP-31g) (kJ/mol) of various species involving the Cp* of complex **1** and its Me_4fulvene adducts interacting with MeOH and MeO, calculated in vacuum.

Fig. S13 E and Z conformations of $[Rh(N,N')(Cp^*\text{-}R)(OH)]^+$ and $[Rh(bipy)(Me_4\text{-}fulv)(OH_2)]^+$.

Fig. S14 Energies and entropy values for the catalytic molecular machine.

References

S.1. Materials and Methods

Rhodium (III) trichloride hydrate was purchased from Precious Metals Online (PMO Pty Ltd.) and used as received. The ligands 3-phenyl-1,2,4,5-tetramethyl-1,3-cyclopentandienyl (Cp^{xPh}) and 3-biphenyl-1,2,4,5-tetramethyl-1,3-cyclopentandienyl (Cp^{xPhPh}) were synthesized following methods in the literature.[1,2] 2,2'-Bpyridine, 1,10-phenanthroline, 4-bromo-biphenyl, n-butyllithium in hexane 1.6 M, phenyllithium in ether (1.8 mM), 2,4-pentamethylcyclopentadiene, 2,3,4,5-tetramethyl-2-cyclopentanone, isoprene, conjugated (9Z,11E)-linoleic acid, α-terpinene, sorbic acid, ethyl sorbate, 3,6-di-2-pyridyl-1,2,4,5-tetrazine and 1,3 diphenylisobenzofuran were obtained from Sigma-Aldrich. Magnesium sulphate and ammonium hexafluorophosphate were obtained from Fisher Scientific. DMSO-d_6, MeOD-d_4, D_2O, acetone-d_6, MeCN-d_3 were purchased from Sigma-Aldrich and Cambridge Isotope Labs Inc. Non-dried solvents used in synthesis were obtained from Fisher Scientific and Prolabo.

^{1}H NMR spectra were acquired in 5 mm NMR tubes at 298 K on either Bruker AV-400 or Bruker AV III 600 spectrometers. For kinetics measurements, spectra were acquired at 310 K unless specified in the text. Data processing was carried out using XWIN-NMR version 3.6 (Bruker U.K. Ltd). ^{1}H NMR chemical shifts were internally referenced to TMS *via* 1,4-dioxane in D_2O ($\delta = 3.66$ ppm). 1D spectra were recorded using standard pulse sequences. Typically, data were acquired with 16, 32 or 128 transients into 32 k data points over a spectral width of 20 ppm, and for the

kinetic experiments, 32 transients into 32 k data points over a spectral width of 20 ppm using a relaxation delay of 2 s.

Positive ion electrospray mass spectra were obtained on a Bruker Daltonics Esquire 2000 ion trap mass spectrometer. All samples were prepared in methanol. Data were processed using Data-Analysis version 3.3 (Bruker Daltonics). HR-MS analysis was carried with a Bruker MaXis plus Q-TOF mass spectrometer equipped with electrospray ionisation source. The mass spectrometer was operated in electrospray positive ion mode with a scan range 50-2,400 m/z. Source conditions were: end plate offset at -500 V; capillary at -4000 V; nebulizer gas (N_2) at 0.5 bar; dry gas (N_2) at 4 L/min; dry temperature at 453 K. Ion tranfer conditions as: ion funnel RF at 200 Vpp; multiple RF at 200 Vpp; quadruple low mass set at 50 m/z; collision energy at 5.0 eV for MS and 10-20 eV for MS/MS, MS/MS isolation window 10; collision RF at 500-2000 Vpp; transfer time set at 50-150 µs; pre-pulse storage time set at 5 µs. Calibration was carried out with sodium formate (10 mM) before analysis.

The x-ray photoelectron spectroscopy (XPS) data were collected at the Warwick Photoemission Facility, University of Warwick. The samples investigated in this study were attached to electrically-conductive carbon tape, mounted on to a sample bar and loaded in to a Kratos Axis Ultra DLD spectrometer which possesses a base pressure below 1 x 10^{-10} mbar.

For XPS measurements, reaction mixtures were used directly without further purification. The solvents (d_4-MeOD/D_2O) were evaporated by freeze-drying. XPS measurements were performed in the main analysis chamber, with the sample being illuminated using a monochromated Al Kα x-ray source (hν = 1486.7 eV). The measurements were conducted at ambient temperature and at a take-off angle of 90° with respect to the surface parallel. The core level spectra were recorded using a pass energy of 20 eV (resolution approx. 0.4 eV), from an analysis area of 300 µm x 700 µm. The work function and binding energy scale of the spectrometer were calibrated using the Fermi edge and $3d_{5/2}$ peak recorded from a polycrystalline Ag sample prior to the commencement

of the experiments. In order to prevent surface charging, the surface was flooded with a beam of low energy electrons throughout the experiment and this necessitated recalibration of the binding energy scale. To achieve this, the main C-C/C-H component of the C 1*s* spectrum was referenced to 284.6 eV. The data were analysed in the CasaXPS package, using Shirley backgrounds and mixed Gaussian-Lorentzian (Voigt) lineshapes. For compositional analysis, the analyser transmission function was determined using clean metallic foils to determine the detection efficiency across the full binding energy range.

S.2. Synthesis and characterization of complexes 1-4

[(Cp*)Rh(bpy)Cl]PF_6 (1): 2,2' bipyridine (35.4 mg, 226 µmol) in dichloromethane (2 mL) was added to [Cp*RhCl_2]$_2$ (49.3 mg, 80.0 µmol) in dichloromethane (5 mL) following our reported procedure.[3] The reaction was stirred for 2.5 h at ambient temperature. NH_4PF_6 (182.6 mg, 1.12 mmol) was then added to the reaction mixture and stirred for 2 h. The crude product was collected by filtration and recrystallized from acetone to obtain [Rh(Cp*)(bpy)Cl] PF_6 as orange crystals (64.8 mg, 112.9 µmol, 50%). ESI-MS: Calc for $C_{20}H_{23}ClN_2Rh$ $(M)^+$ *m/z* = 429.06 found *m/z* = 429.2. ^{1}H-NMR (400 MHz, acetone-D_6): δ = 9.18 (d, *J* = 5.3 Hz, 2H, bpy-H6), 8.70 (d, J = 8.0 Hz 2H, bpy-H3), 8.41 (t, *J* = 7.9 Hz, 2H, bpy-H5), 7.98 (t, *J* = 6.5 Hz 2H, bpy-H4), 1.85 (s, 15H, CH_3) ppm.

[(Cp*)Rh(phen)Cl]PF_6 (2): Synthesis was carried out following our reported protocol.[3] A dichloromethane solution (2 mL) of 1,10-phenanthroline·H_2O (42.2 mg, 212 µmol) was added to a dichloromethane solution (8 mL) of [Cp*RhCl_2]$_2$ (49.6 mg, 80.2 µmol). The reaction was stirred for 1.5 h at room temperature. NH_4PF_6 was then added in excess and the mixture was stirred for 1 h. The crude product was collected by filtration and re-crystallized from acetone to obtain [Rh(Cp*)(phen)Cl]PF_6 as orange crystals (82.4 mg, 137.8 µmol, 65%). ESI-MS: Calc for $C_{22}H_{23}ClN_2Rh$ $(M)^+$ *m/z* = 453.1 found *m/z* = 453.2. ^{1}H-NMR (400 MHz, acetone-d_6): δ = 9.54 (d,

J = 4.2 Hz, 2H, phen-H2), 8.96 (d, J = 8.2 Hz, 2H, phen-H4), 8.33 (s, 2H, phen-H5), 8.27 (t, J = 6.2 Hz, 2H, phen-H3), 1.88 (s, 15 H, CH_3) ppm.

[(Cp^{xPh})Rh(phen)Cl]PF_6 (3): Synthesis was carried out following our reported protocol.[3] 1,10-Phenanthroline·H_2O (36.6 mg, 185 µmol) in dichloromethane (2 mL) was added to dichloromethane solution (8 mL) of [(Cp^{xPh})$RhCl_2$]$_2$ (50.3 mg, 68.0 µmol). The reaction mixture was stirred for 2 h at room temperature followed by addition of NH_4PF_6 (110.8 mg, 680 µmol) and the resultant mixture was stirred for 1 h. The crude product was collected by filtration and re-crystallized from acetone to obtain [Rh(Cp^{xPh})(phen)Cl]PF_6 as orange crystals (81.8 mg, 124.0 µmol, 67%). ESI-MS: Calc for $C_{27}H_{25}ClN_2Rh$ (M)+ m/z = 515.08 found m/z = 515.2.^{1}H-NMR (400 MHz, acetone-d_6): δ = 9.20 (d, J = 4.3 Hz, 2H, phen-H2), 8.97 (d, J = 8.4 Hz, 2H, phen-H4), 8.35 (s, 2H, phen-H5), 8.19 (t, J = 6.2 Hz, 2H, phen-H3), 7.84 (d, J = 7.4 Hz, 2H,Cp^{xPh}-H2/H6), 7.68-7.55 (m, 3H, Cp^{xPh}-H3/H4/H5), 1.98 (s, 6H, CH_3, Cp^{xPh}-H3'/H4'), 1.89 (s, 6H, CH_3, Cp^{xPh}-H2'/H5') ppm.

[(Cp^{xPhPh})Rh(phen)Cl]PF_6 (4): 1,10-Phenanthroline·H_2O (29.1 mg, 147 µmol) in dichloromethane (2 mL) was added to dichloromethane solution (8 mL) of [(Cp^{xPhPh})$RhCl_2$]$_2$ (48.6 mg, 54.5 µmol). The reaction mixture was stirred for 1.5 h at room temperature. NH_4PF_6 was then added in excess and the resultant reaction mixture was stirred for 1 h. The crude product was collected by filtration and re-crystallized from acetone to obtain [Rh(Cp^{xPhPh})(phen)Cl] PF_6 as orange crystals (57.3 mg, 77.9 µmol, 53 %). ESI-MS: Calc for $C_{33}H_{29}ClN_2Rh$ $(M)^+$ m/z = 591.1 found m/z = 591.2. ^{1}H-NMR (400 MHz, $CDCl_3$): δ = 8.93 (d, J = 4.2 Hz, 2H, phen-H2), 8.61 (d, J = 7.3 Hz, 2H, phen-H4), 8.07 (s, 2H, phen-H5), 7.99 (t, J = 5.9 Hz, 2H, phen-H3), 7.87 (d, J = 6.2 Hz, 2H,Cp^{xPhPh}-H2'/H6'), 7.83 (d, J = 5.5 Hz, 2H, Cp^{xPhPh}-H3'/H5'), 7.71 (d, J = 8.2 Hz, 2H, Cp^{xPhPh}-H2/H6), 7.53 (t, ^{J} = 7.5 Hz 2H, Cp^{xPhPh}-H3/H5), 7.46 (m, 1H, Cp^{xPhPh}-H6), 1.90 (s, 6H, CH_3, Cp^{xPhPh}-H2''/H5''), 1.85 (s, 6H, CH_3, Cp^{xPhPh}-H3''/H4'')ppm.

S.3. Diels-Alder reactions

Complex **5** (isoprene adduct)

To study the [4+2] Diels-Alder reactions between complex **1** and isoprene, 4.36 µL isoprene (2.96 mg, 43.6 µmol) was added to [Cp*Rh(bpy)Cl] PF_6 (**1**) (2.50 mg, 4.36 µmol) in 1.56 mL

d_4-MeOD/D_2O (3:2) or in 1.56 mL d_4-MeOD in the dark following our reported protocol.[3] The reaction was stirred for 12 h at ambient temperature. Then the reaction mixture was freeze-dried immediately, or the solvents were evaporated. The product was characterized by LCMS, HRMS and XPS.

XPS yield in the crude reaction mixture: 50.0 %; HRMS: Calculated m/z for [**5**-Cl]$^+$; $C_{25}H_{30}N_2Rh$ = 461.1459, Observed: 461.1496; LC-MS: 461.08; ESI-MS: 461.2. **^{1}H-NMR** (400 MHz, d_6-acetone): 8.75 (m, 4H), 8.31 (d, 7.99 Hz, 2H), 7.85 (t, 6.27 Hz, 2H), 5.57 (m, 1H), 1.84 (s, 6H), 1.83 (s, 6H) ppm.

Complex **6** (α-terpinene adduct)

In the dark, 7.00 µL of α-terpine (5.86 mg, 43.0 µmol) was reacted with [(Cp*)Rh(bpy)Cl] PF_6(**1**) (2.50 mg, 4.36 µmol) in 1.56 mL d_4-MeOD for 12 h at ambient temperature to study the [4+2] adduct formation between **1** and α-terpine. The solvents were evaporated. The product was characterized by HRMS and XPS.

XPS yield in the crude reaction mixture: 12.5 %; HRMS: Calculated m/z for [**6**-Cl]$^+$; $C_{30}H_{38}N_2Rh$ = 529.2085, found *m/z* = 529.2113.

Complex **7** (ethyl sorbate adduct)

Complex **7** was synthesized from a [4+2] Diels-Alder reaction between the ethyl sorbate (7.38 mg, 52.6µmol) and [Cp*Rh(bpy)Cl] PF_6 (**1**) (2.84 mg, 4.95 µmol) in 1.77 mL d_3-MeCN/d_4-MeOD (3:2). The reaction was carried out in the dark at ambient temperature over 12 h. The solvents were evaporated. The product was characterized by HRMS, LCMS and XPS.

HRMS: Calculated m/z for [**7**-Cl]$^+$; $C_{28}H_{34}N_2O_2Rh$ = 533.1670, found *m/z* = 533.1664

Complex **8** ((9*Z*,11*E*) conjugated linoleic acid adduct)

To study the Diels-Alder reactions between complex **1** and (9*Z*,11*E*) conjugated linoleic acid, in the dark, (9*Z*,11*E*) conjugated linoleic acid (10.8 mg, 38.5 µmol, 10.0 eq) was added to

[(Cp*)Rh(phen)Cl] PF_6 (**1**) (2.21 mg, 3.85 µmol, 1.00 eq) in 1.38 mL d_4-MeOD following our reported protocol.[3] The reaction was stirred overnight at room temperature. The solvents were evaporated. The product was characterized by LCMS, HRMS and XPS.

HRMS**:** Calculated m/z for [**8**-Cl]$^+$; $C_{38}H_{54}N_2O_2Rh^+$ = 673.3235, found *m/z* = 673.3229. LCMS: 691.21 [M+H_2O]$^+$.

S.4. DFT calculations

Applied methods

The calculations were performed using Gaussian 16 package with use of the CAM-B3LYP functional and CEP-31g or QZVP basis set (see text). IEFPCM method was used to model the methanol solvent. The corresponding structures of the reactants and products were optimized and the frequency calculations were subsequently performed in order to show that the true minimum was achieved. The saddle (transition) points were calculated using the quadratic synchronous transit method (QST3 keyword of Gaussian) with calculations of the forces (CalcAll keyword of Gaussian). The subsequent frequency calculations were performed revealing one negative frequency mode in case of both models (with and without methanol molecule). Finally, the internal reaction coordinate (IRC) calculations were performed that additionally confirmed the transition state character of the saddle point found within the QST3 approach.

E- and Z-isomerism in complexes of monosubstituted R-Cp(Me)$_4$ complexes

For the Cp*-substituted ligand systems under considerations, two conformations of the complex are possible, with the phenyl or biphenyl substituent located either on the side of N,N'-Rh plane with coordinated oxygen ligand, or on the opposite one, resulting in E and Z conformers for details. These conformers are illustrated in **Fig. S13**. They may be described as E and Z conformers with respect to the O-Rh-Cp*-R fragments. The isomers are shown in **Fig. S13**. Note that this isomerism occurs also for the square-planar species.

Modelling the reactivity of the catalyst precursor towards for forming hydroxide-species

In order to obtain insight into possible sources of this dependence, other than the electronic effect of the ligands and substituents we examined the reactivity of the catalyst precursors [Rh(N,N')(Cp*-R)Cl]$^+$ towards forming the hydroxido-species [Rh(N,N')(Cp*-R)(OH)]$^+$. We modelled the process of the subsequent aquation of the precatalyst followed by the deprotonation of the coordinated water leading to formation of the hydroxido-species. The following reaction was therefore modelled:

$[Rh(N,N')(Cp^*\text{-}R)Cl]^+ + 2H_2O \rightarrow [Rh(N,N')(Cp^*\text{-}R)(H_2O)]^{2+} + H_2O + Cl^- \rightarrow [Rh(N,N')(Cp^*\text{-}R)(OH)]^+ + H_3O^+ + Cl^-$.

For the sake of simplicity, we excluded the methanol molecule from the model. As the previously mentioned calculation for the substituted Cp* ligands show that the E and Z conformers of all investigated stages of the proton transfer differ by less than 3 kJ/mol in energy, we performed the calculations for the aquation and deprotonation process only for the E conformations of the Cp*-Ph and Cp*-biph complexes. The results are collected in **Table S2** and shown schematically in **Fig. 13**. Due to the general problem of estimation of the explicit solvent interaction with DFT mentioned above and discussed in ref. 4, the absolute energies shall be treated as the estimates.

Table S1. Calculated (CAM-B3LYP/QZVP) Rh-$N_{diimine}$ distances (Å) for $[Rh(N,N')(Cp^*\text{-}R)OH]^+ \cdot MeOH$ $[Rh(N,N')(Me_4\text{-}fulv)OH_2]^+ \cdot MeOH$ and square planar $[Rh(N,N')(Me_4\text{-}fulv)]^+ \cdot MeOH \cdot H_2O$ systems.

Diamine/Cp*-R	Axial ligand OH	Axial ligand H_2O	Square-planar species[a]
bipy/Cp*	2.113, 2.115	2.130,2.137	2.094
phen/Cp*	2.129, 2.126	2.152, 2.139	2.107, 2.105
phen/Z-Ph	2.118, 2.121	2.142	2.102, 2.099
phen/E-Ph	2.125	2.134, 2.154	2.104, 2.107
phen/Z-biph	2.115, 2.126	2.143, 2.145	2.105, 2.102
phen/E-biph	2.124, 2.125	2.135, 2.155	2.107, 2.103

[a]Square-planar coordination of Rh with only diimine and Cp*-fulvene ligands bound

Table S2. Calculated estimated relative energies (kJ/mol) of the stages of the aquation/deprotonation of the $[Rh(N,N')(Cp^*\text{-}R)Cl]^+$ complexes calculated with CAM-B3LYP/QZVP with methanol as solvent.

**System**	**Interacting species/Energy (kJ/mol)**		
**N,N' ligand/Cp*-R conformation[a]**	$[Rh(N,N')(Cp^*\text{-}R)Cl]^+ + 2H_2O$	$[Rh(N,N')(Cp^*\text{-}R)H_2O]^{+2} + H_2O + Cl^-$	$[Rh(N,N')(Cp^*\text{-}R)OH]^+ + H_3O^+ + Cl^-$

bipy, Cp*	0	23	146
phen, Cp*	0	15	128
phen,E-Cp*biph	0	16	123
phen, E-Cp*-Ph	0	15	122

[a] For simplicity of labelling, Cp*-R = Me_4Cp-R, where R = phenyl or biphenyl

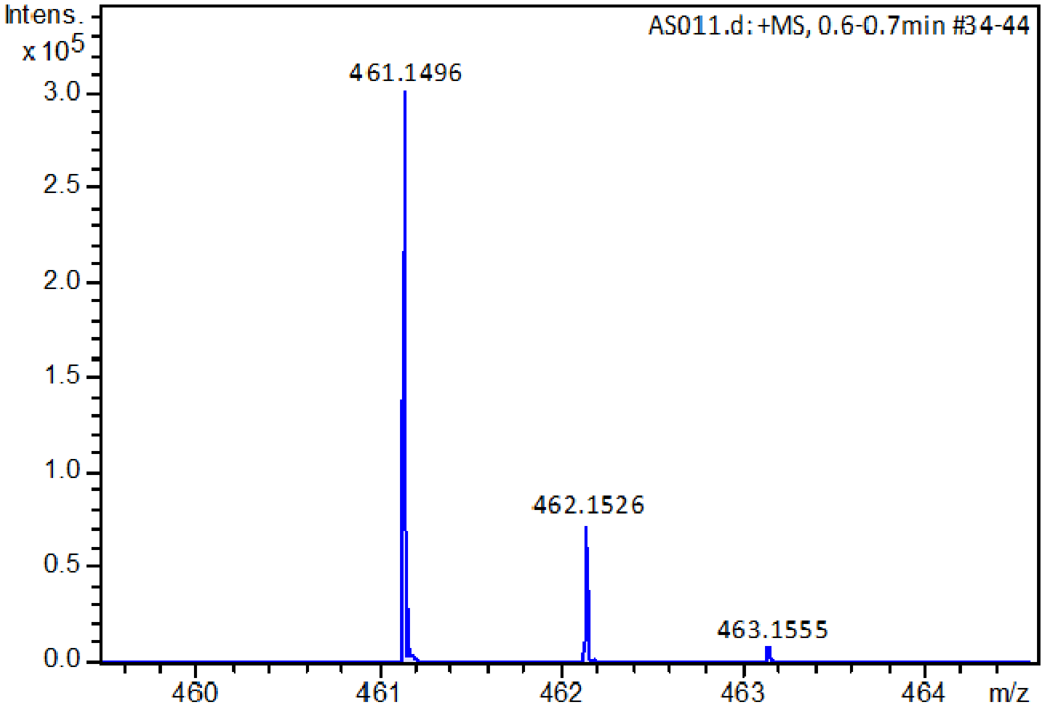

Fig. S1 HRMS characterization of the isoprene Diels-Alder adduct, complex **5**. The peak at m/z = 461.1496 corresponds to [**5**-Cl]$^+$ (calc. m/z = 461.1459).

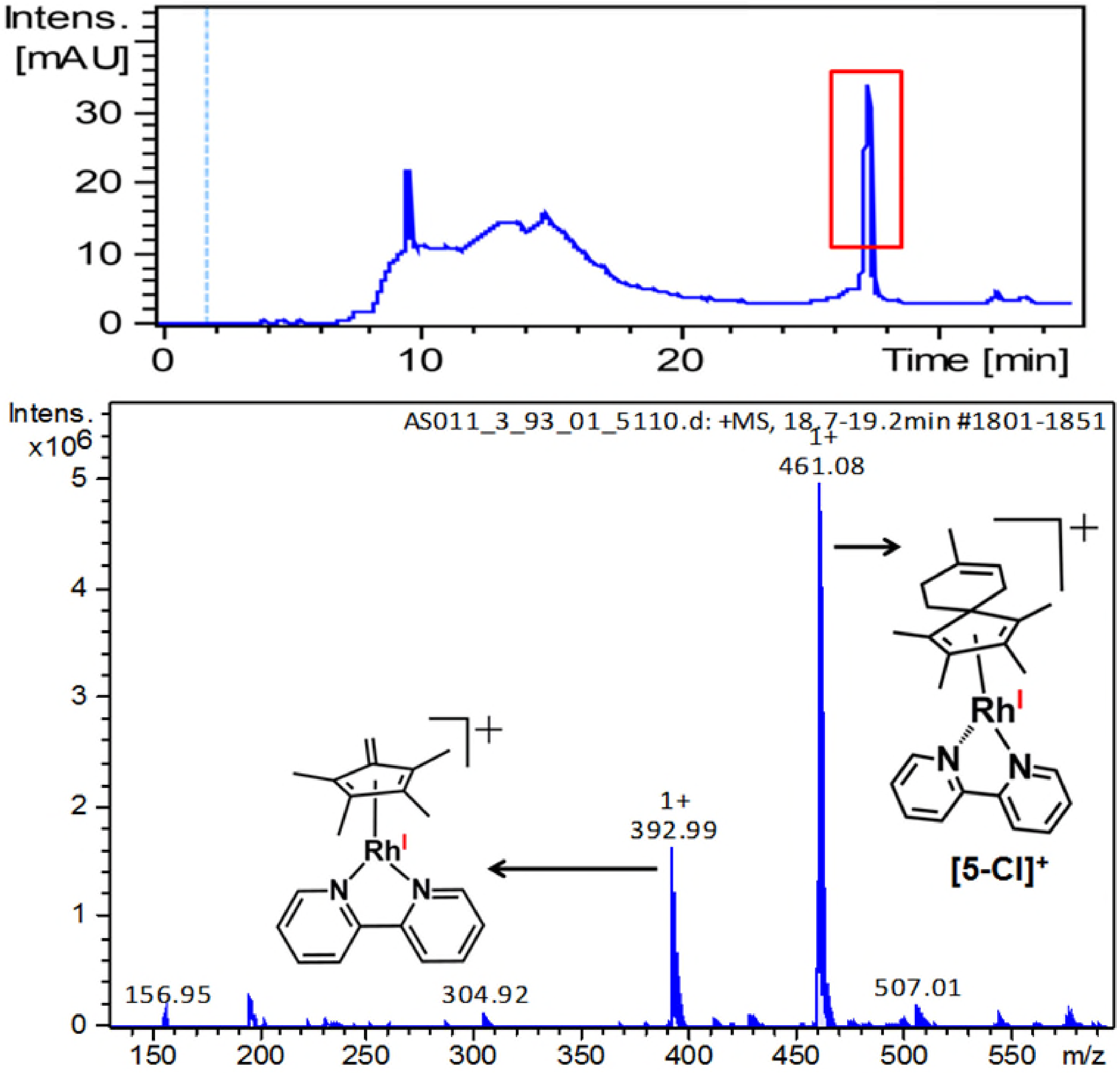

Fig. S2 LC-MS characterization of the isoprene adduct, complex **5.** LC-MS was performed on an Agilent 1260 HPLC connected to a Bruker Amazon X instrument using a ZORBAX Eclipse XDB-C18 column (Agilent, 250 × 4.6 mm, 5 μm) at a 1 mL/min flow rate and using 0.1% formic acid in water (A) and 0.1% formic acid in acetonitrile (B) as mobile phases. The following gradient was used: 100% A at 0 min, 95% A t 5 min, 0% A at 25 min, 95% A at 30 min.

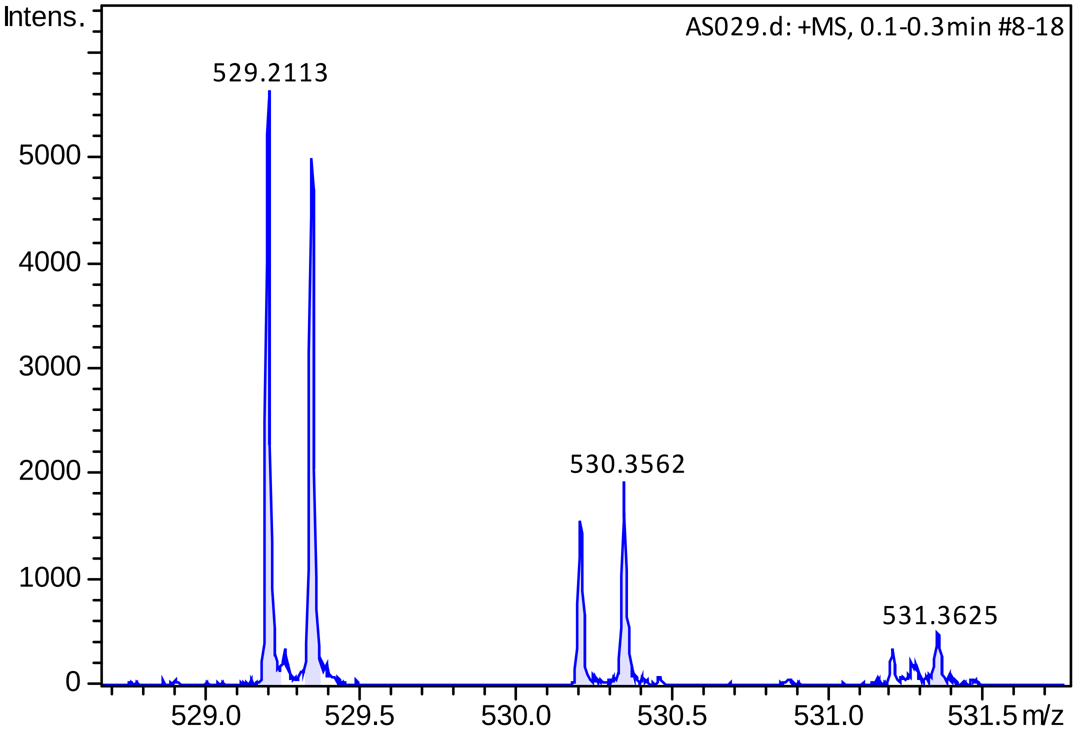

Fig. S3 Characterization of the Diels-Alder adduct of complex **1** with α-terpinene (complex **6**) by HRMS analysis**.** The peak at m/z = 529.2113 corresponds to $C_{30}H_{38}N_2Rh$ = [**6**-Cl]$^+$ (calculated *m/z* = 529.2085).

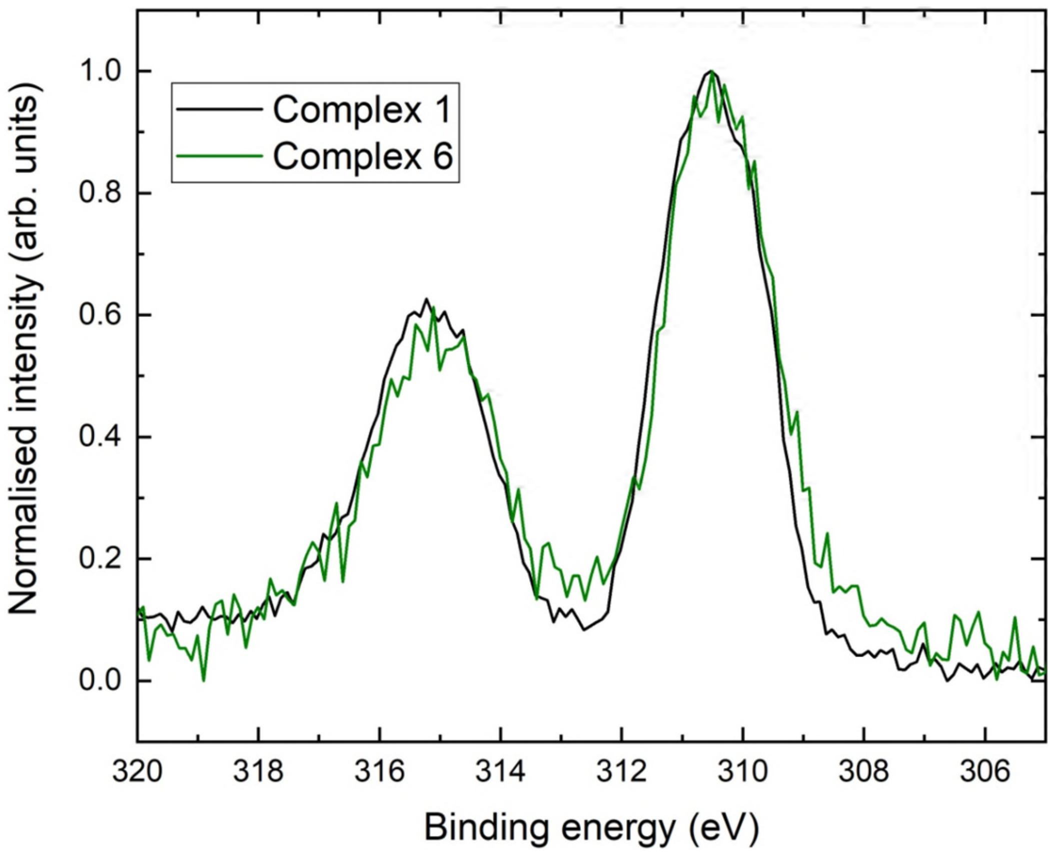

Fig. S4 Normalised Rh 3*d* XP spectra from complexes **1** and **6**, showing a downward shift and broadening of the envelope upon reduction of Rh(III) to Rh(I).

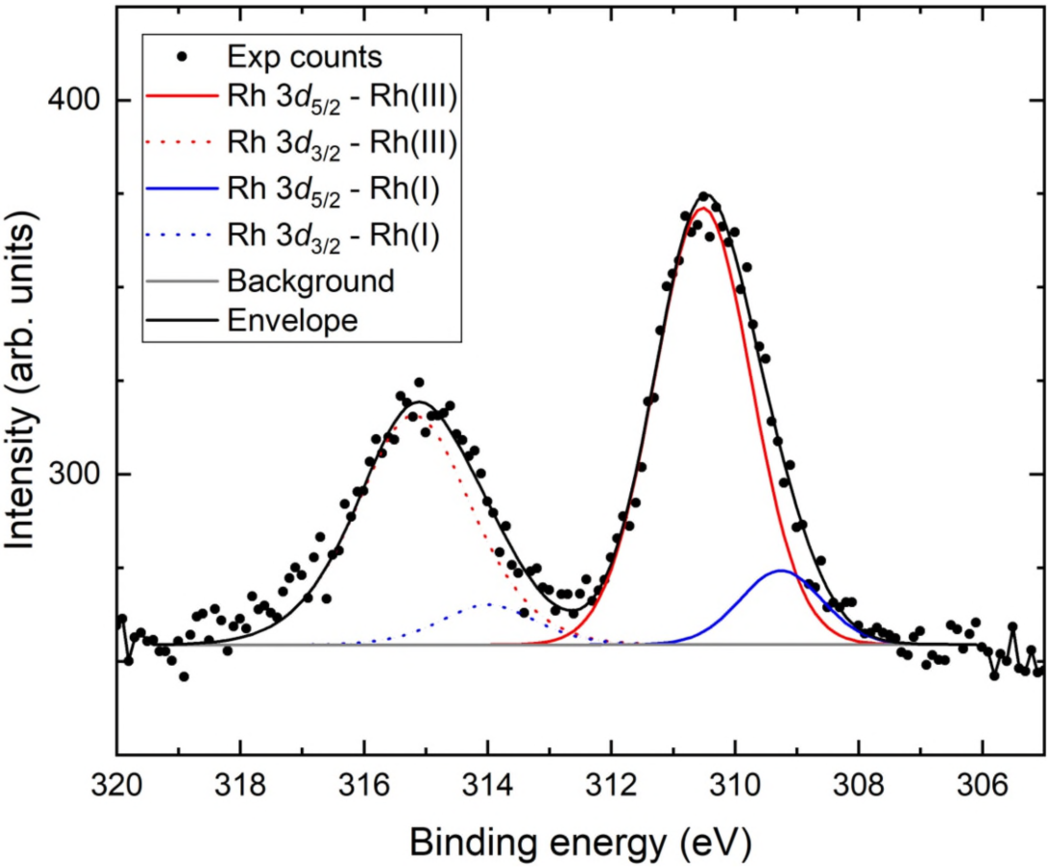

Fig. S5 The Rh XP 3*d* region acquired from complex **6**, illustrating the formation of Rh(I).

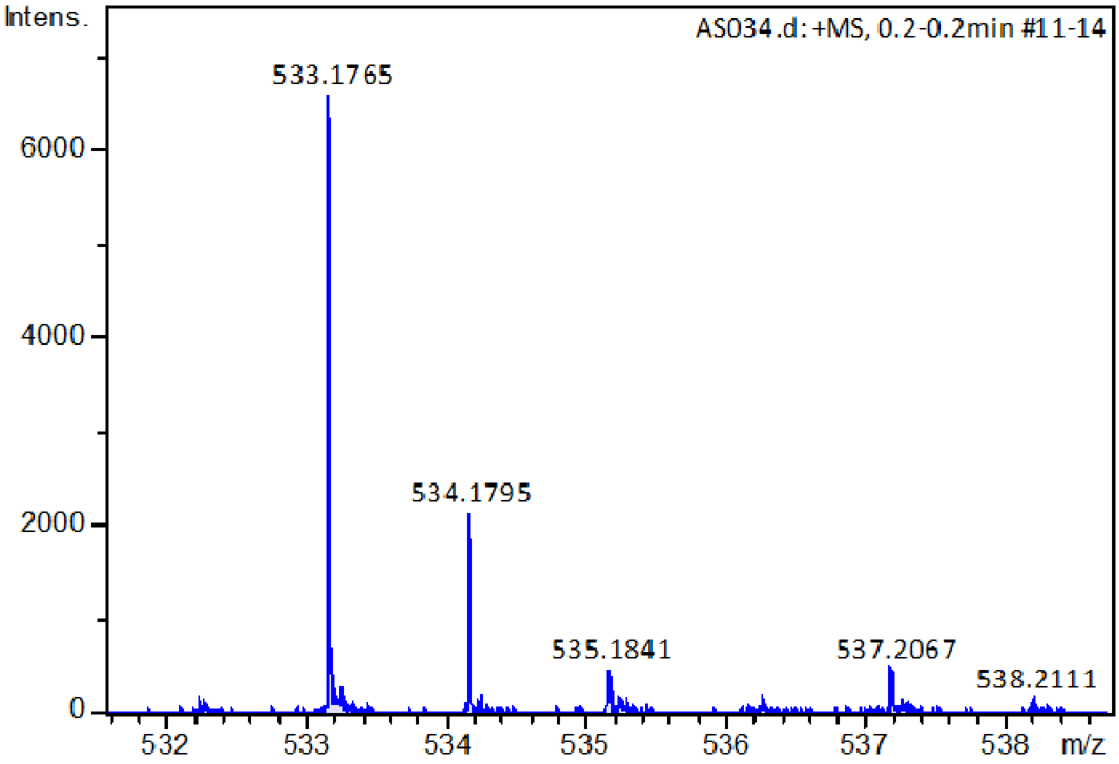

Fig. S6 HRMS characterization of the Diels-Alder adduct of complex **1** with ethyl sorbate (complex **7**)**.** The peak at m/z = 533.1765 corresponds to the $C_{28}H_{34}N_2O_2Rh$ = [**7**-Cl]$^+$ (calculated m/z = 533.1675).

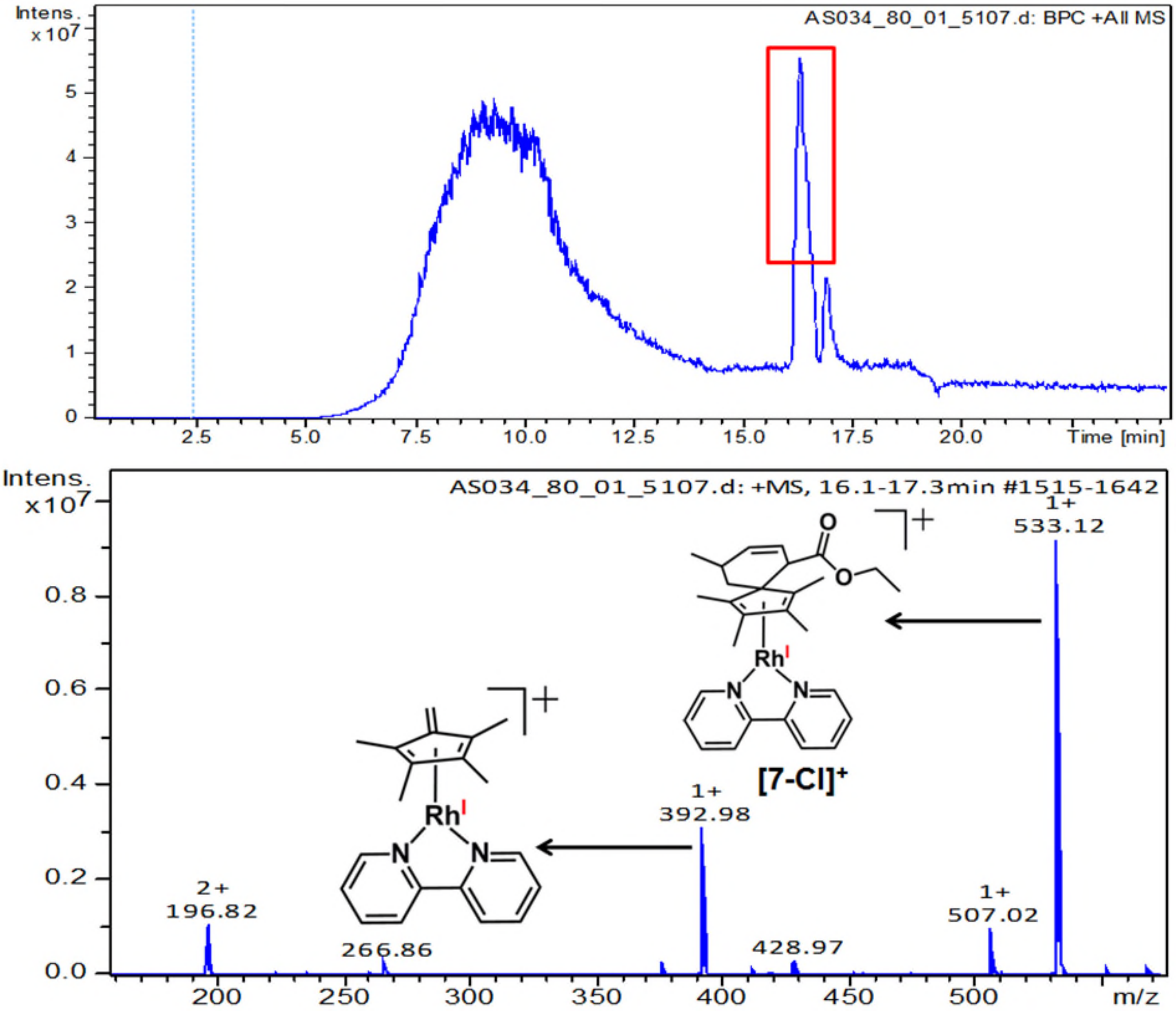

Fig. S7 LC-MS characterization of the ethylsorbate adduct, complex **7**. LC-MS was performed on an Agilent 1260 HPLC connected to a Bruker Amazon X instrument using a ZORBAX Eclipse XDB-C18 column (Agilent, 250 × 4.6 mm, 5 μm) at a 1 mL/min flow rate and using 0.1% formic acid in water (A) and 0.1% formic acid in acetonitrile (B) as mobile phases. The following gradient was used: 100% A at 0 min, 95% A t 5 min, 0% A at 25 min, 95% A at 30 min.

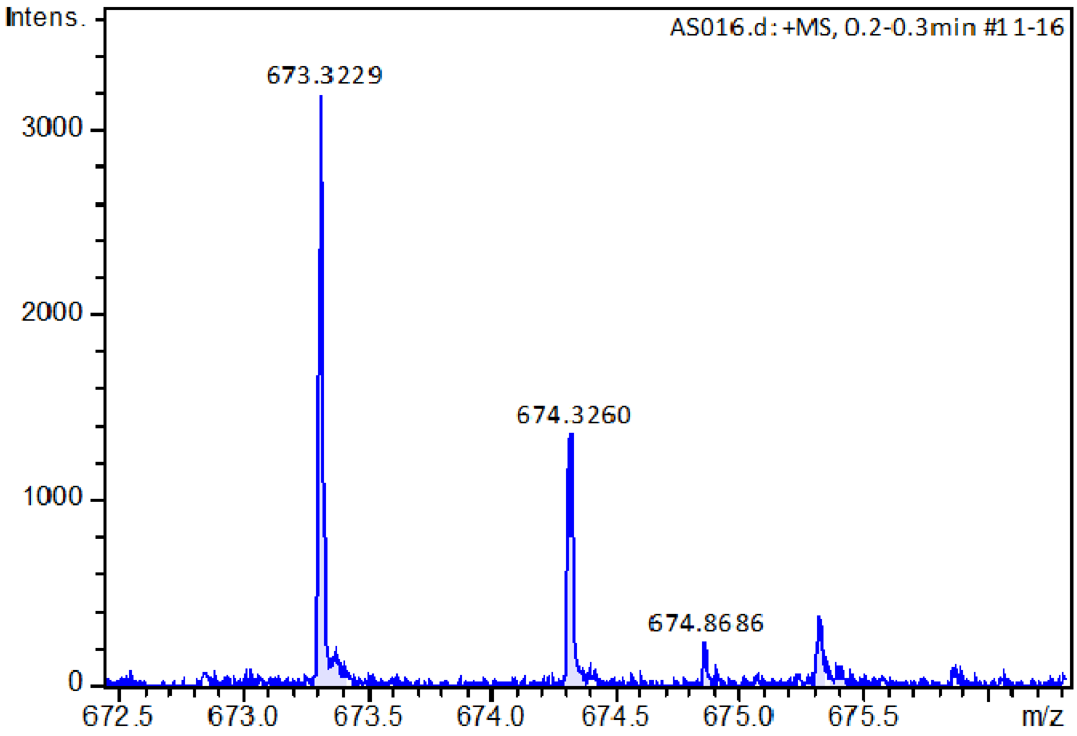

Fig. S8 HRMS characterization of the Diels-Alder adduct of complex **1** with (9*Z*,11*E*) conjugated linoleic acid (complex **8**). The peak at m/z = 673.3229 corresponds to the $C_{38}H_{54}N_2O_2Rh$ = [**8**-Cl]$^+$ (calculated m/z = 673.3240).

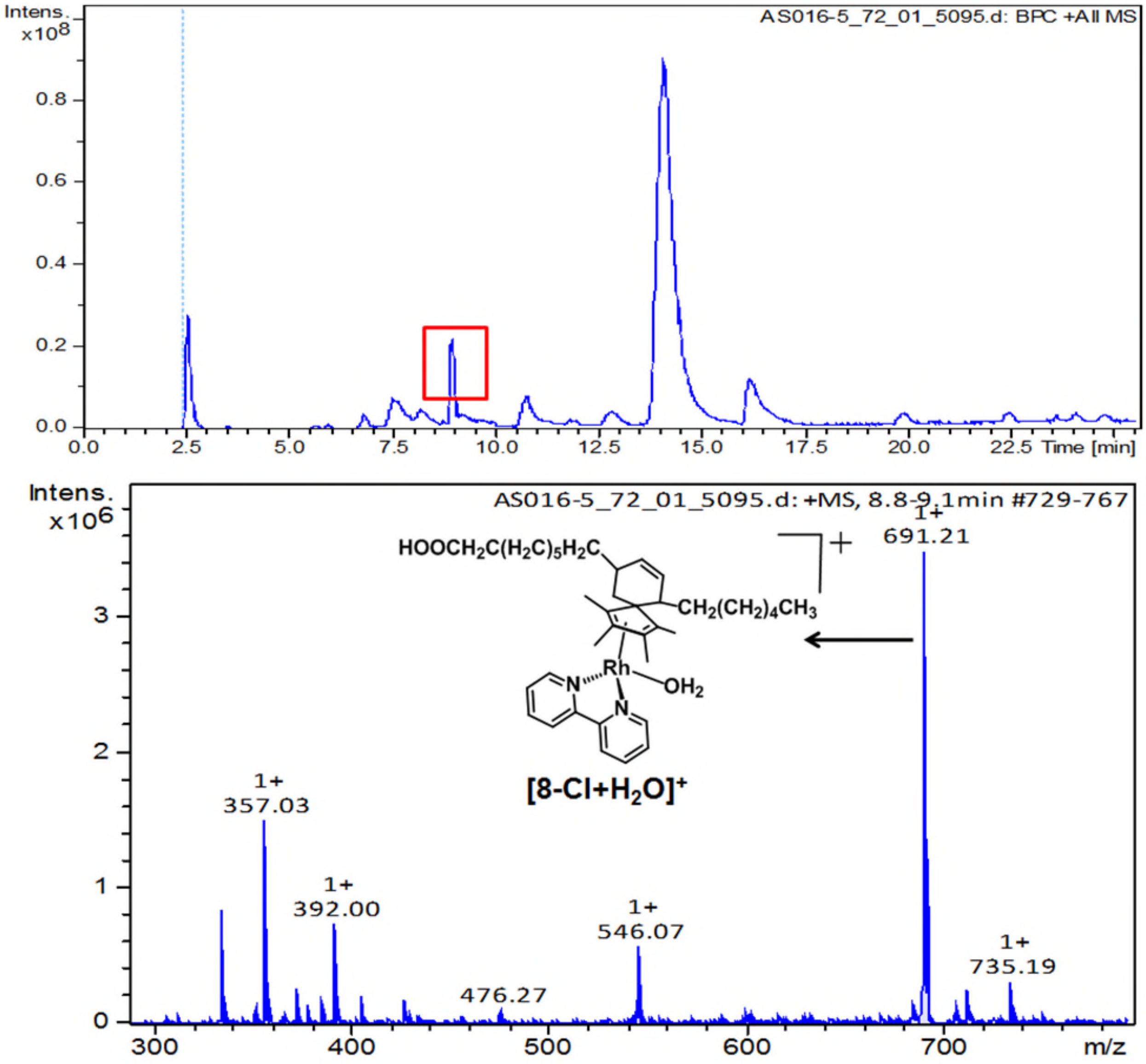

Fig. S9 LC-MS characterization of the conjugated (9*Z*,11*E*)-linoleic acid adduct, complex **8** (calculated m/z for [**8**-Cl+H_2O]$^+$ = 691.33). LC-MS was performed on an Agilent 1260 HPLC connected to a Bruker Amazon X instrument using a ZORBAX Eclipse XDB-C18 column (Agilent, 250 × 4.6 mm, 5 µm) at a 1 mL/min flow rate and using 0.1% formic acid in water (A) and 0.1% formic acid in acetonitrile (B) as mobile phases. The following gradient was used: 100% A at 0 min, 95% A t 5 min, 0% A at 25 min, 95% A at 30 min.

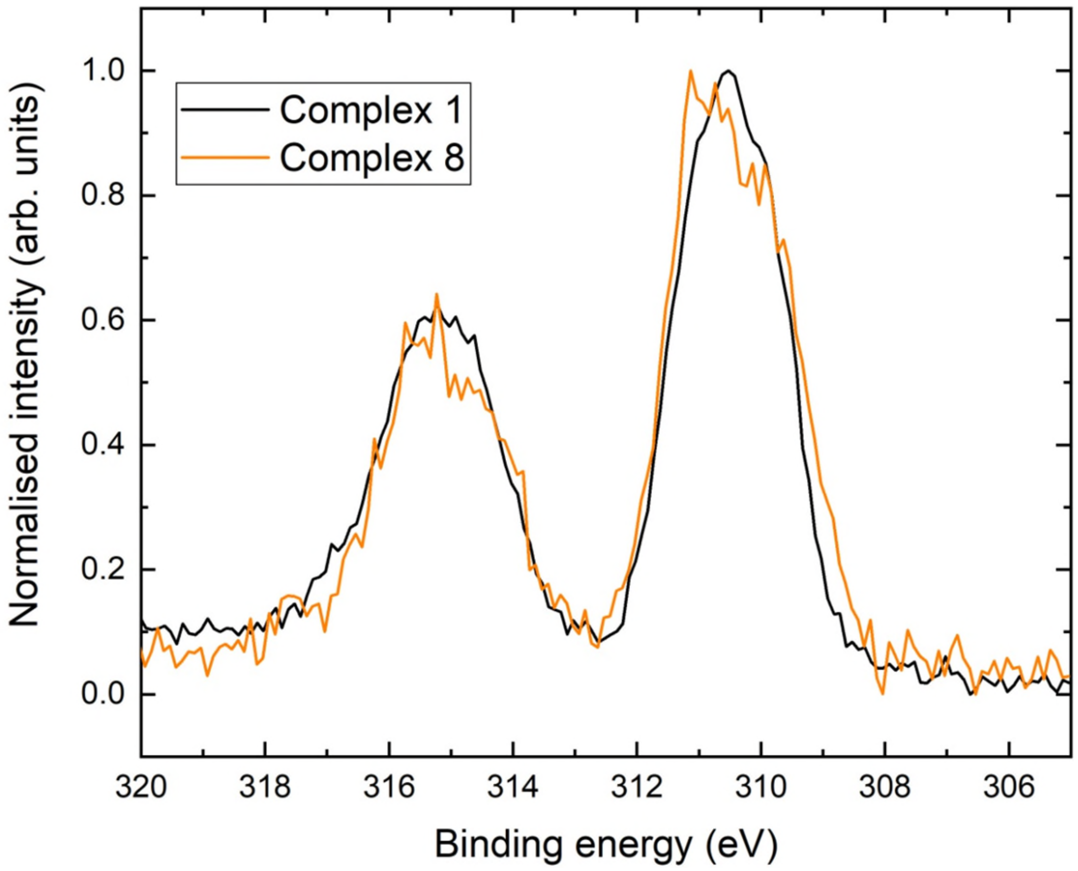

Fig. S10 Normalised Rh 3*d* XP spectra from complexes **1** and **8**, showing a downward shift and broadening of the envelope upon reduction of Rh(III) to Rh(I).

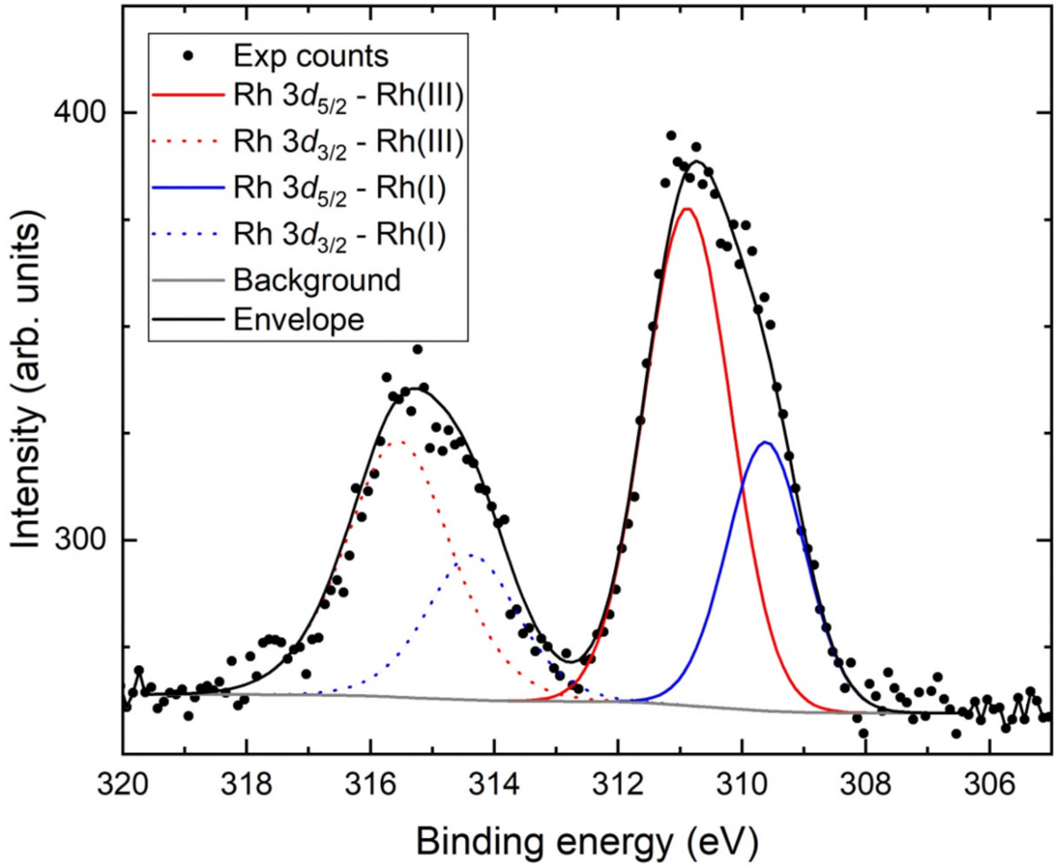

Fig. S11 The Rh XP 3*d* region acquired from complex **8**, illustrating the formation of Rh(I).

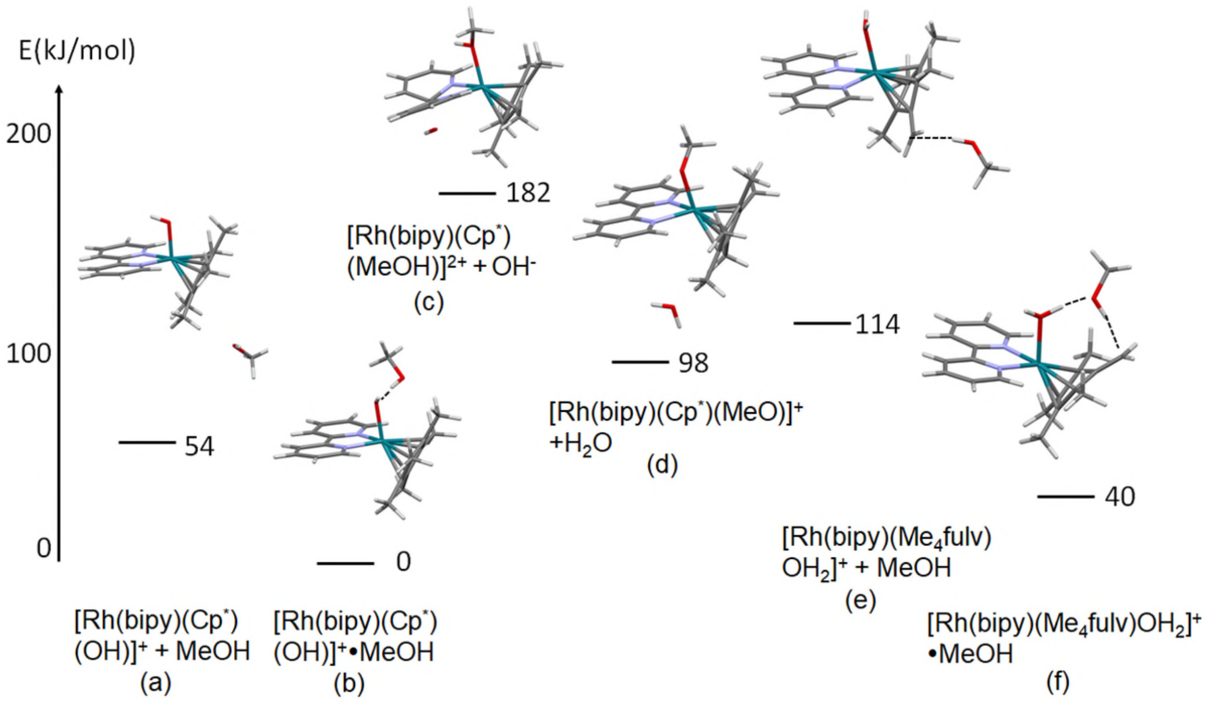

Fig. S12 Calculated electronic energies (CAM-B3LYP/CEP-31g) (kJ/mol) of various species involving the Cp* of complex **1** and its Me_4fulvene adducts interacting with MeOH and MeO^-, calculated in vacuum. Note that the diamine ligand is shown always on the left of the complex, while the Cp*/Cp*-fulvene is on the right. The weak contacts H**O---H**(O_{MeOH}), **H**($(OH)_{MeOH}$)----**C**((CH_2=) $_{Cp}$*) and **H**(water)**---O**(MeOH) are shown with dashed lines. Atom colour code: Rh – turquoise, N- blue, O -red, C- black, H –white/grey.

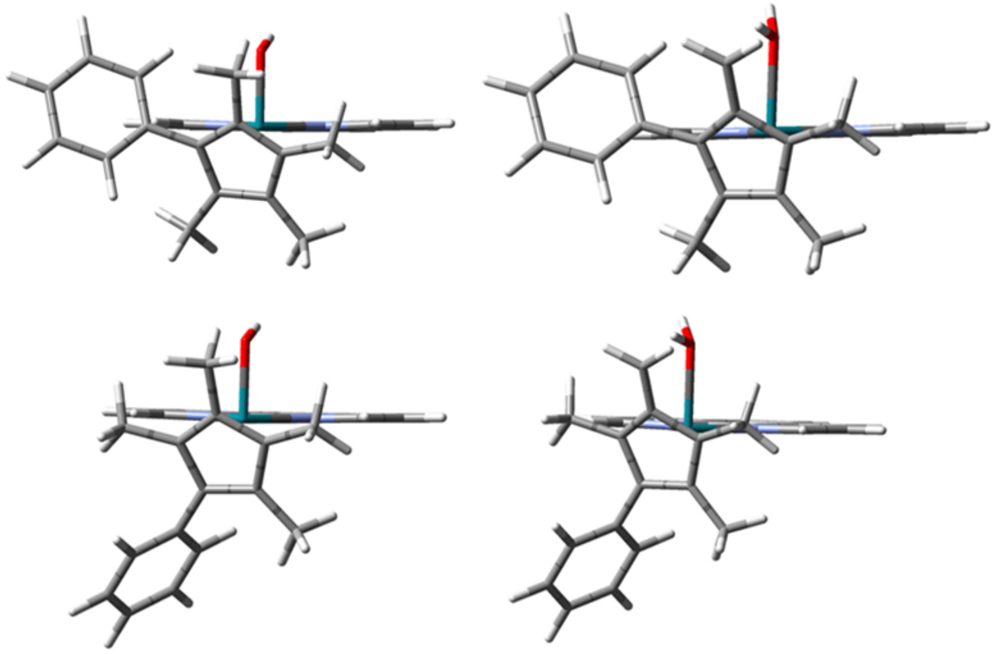

Fig. S13 **Bottom:** E, and **top**: Z conformations of $[Rh(N,N')(Cp^{*}\text{-}R)(OH)]^{+}$ (left) and $[Rh(bipy)(Me_4\text{-}fulv)(OH_2)]^{+}$ (right) species. Note that the *N*,*N*'-diimine ligand is shown in the background with its plane perpendicular to the plane of the figure. Colour code: Rh – turquoise, N- blue, O -red, C- black, H –white/grey.

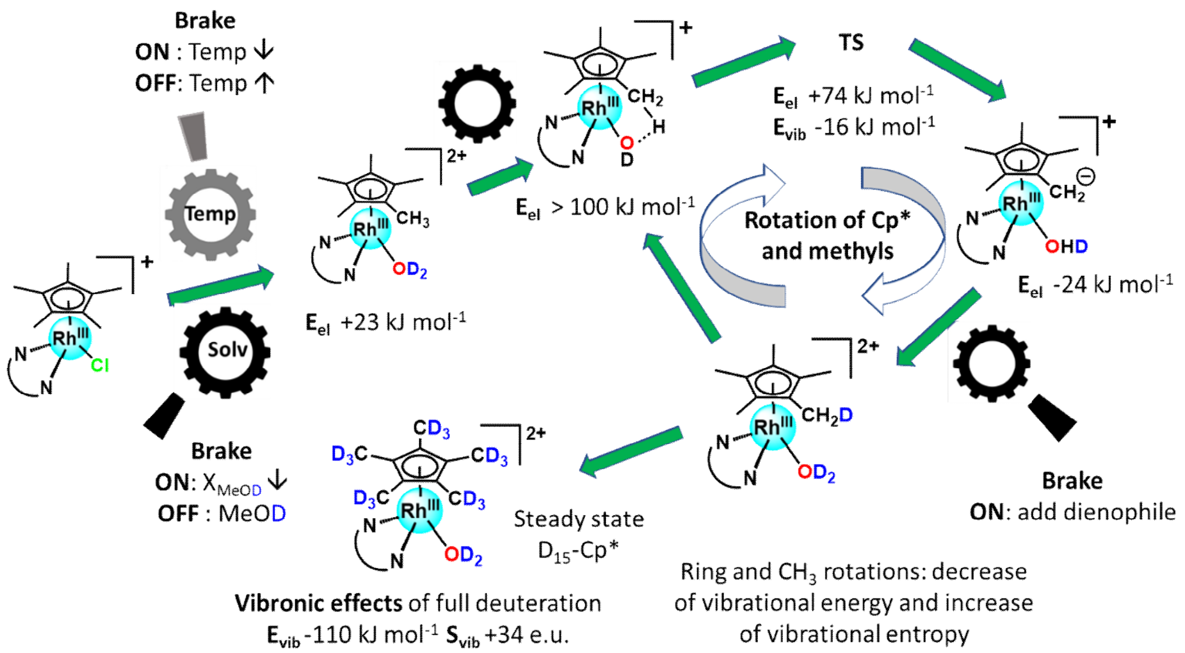

Fig. 14 Electronic and vibrational energies, and entropy values, for the catalytic molecular machine, based on the data in this paper and ref. 5. The diagram illustrates the switching-on of the pre-catalyst by hydrolysis, deprotonation and activation of coordinated water, control of the rate of reaction by temperature or solvent changes, deprotonation of a ring methyl, formation of a Rh(I) fulvene intermediate, addition of a dienophile to form a Diels-Alder adduct which stalls the machine, rotation of the Cp* ring and methyls, which in 14 further cycles leads to complete sequential deuteration of the Cp* ligand. It is notable that vibrational effects make a significant contribution to the overall driving force for the reactions. Overall vibronic effects are estimated for the hydroxide species (ref. 5).

References

[1] M. Björgvinsson, S. Halldorsson, I. Arnason, J. Magull and D. Fenske, *J. Organomet. Chem.*, 1997, **544**, 207-215.

[2] Z. Liu, A. Habtemariam, A. M. Pizarro, S. A. Fletcher, A. Kisova, O. Vrana, L. Salassa, P. C. A. Bruijnincx, G. J. Clarkson, V. Brabec and P. J. Sadler, *J. Med. Chem.*, 2011, **54**, 3011-3026.

[3] S. Banerjee, J. J. Soldevila-Barreda, J. A. Wolny, C. A. Wootton, A. Habtemariam, I. Romero-Canelón, F. Chen, G. J. Clarkson, I. Prokes, L. Song, P. B. O'Connor, V. Schünemann and P. J. Sadler, *Chem. Sci.*, 2018, **9**, 3177-3185.

[4] H. Ryu, J. Park, H. K. Kim, J. Y. Park, S.-T. Kim and M.-H. Baik, *Organometallics*, 2018, **37**, 3228−3239.

[5] J. Armstrong, S. Banerjee, V. Schünemann, J. A. Wolny and P. J. Sadler, *J. Phys. Chem. Lett.*, 2021, **12**, 658–662.
