# Supplementary figures and images for: Kinetics and mechanism of sequential ring methyl C–H activation in cyclopentadienyl rhodium(iii) complexes
Source: Dalton Trans. 2022 Aug 31;51(42):16070–81. doi: 10.1039/d2dt02079c (PMC9623609; doi:10.1039/d2dt02079c)

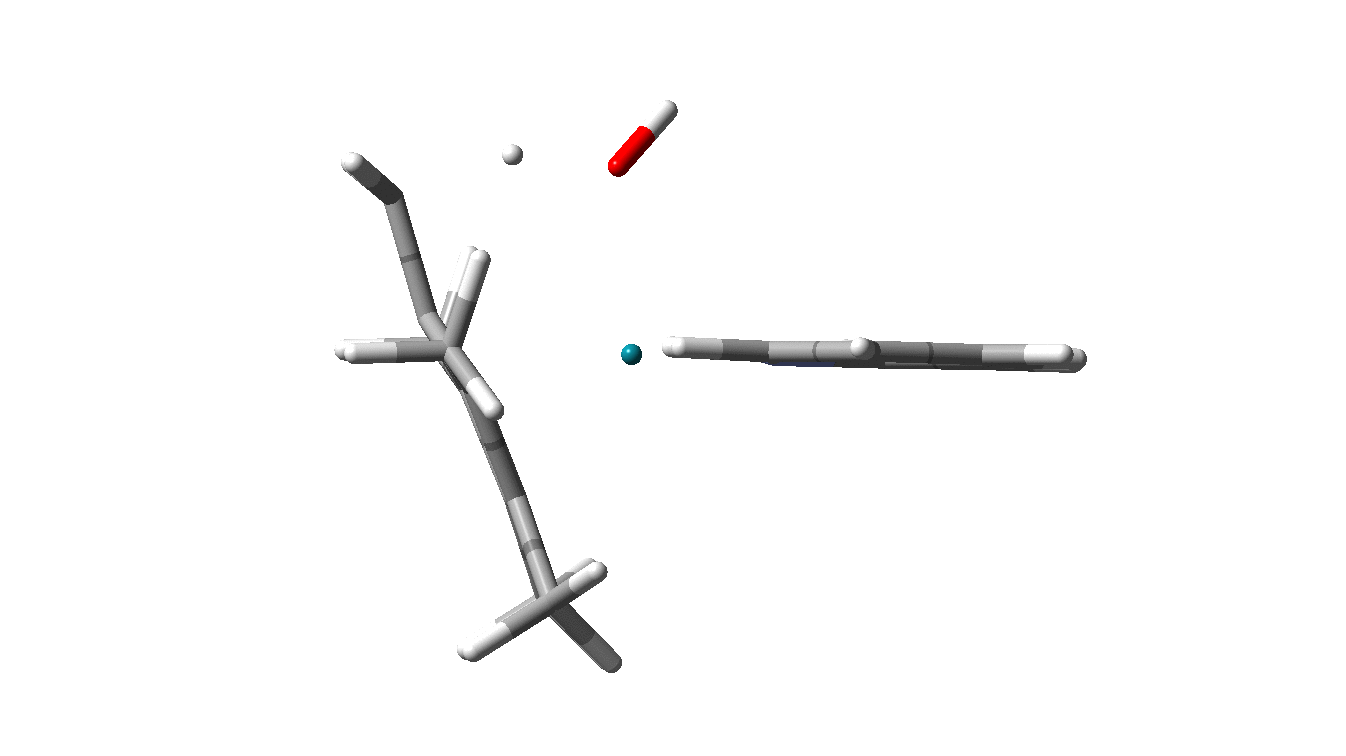

Supplement: DT-051-D2DT02079C-s002 [file DT-051-D2DT02079C-s002.gif]

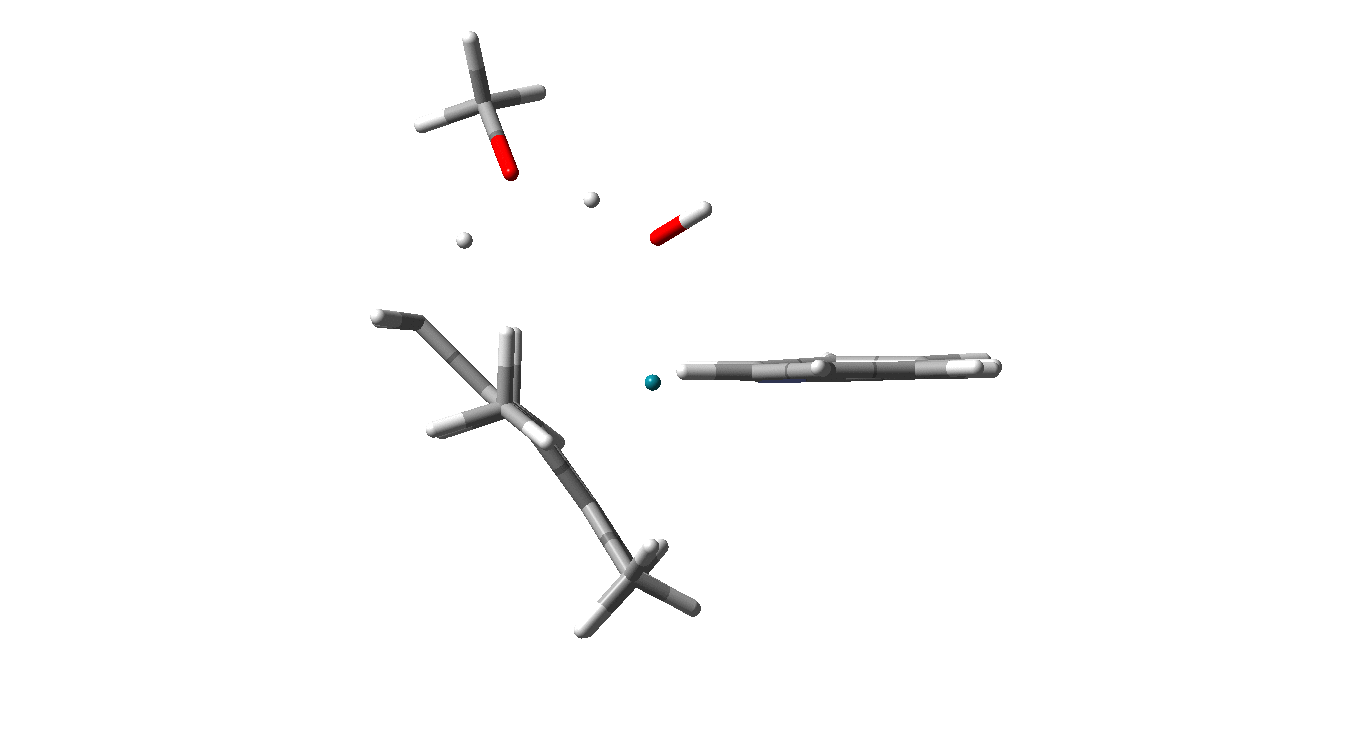

Supplement: DT-051-D2DT02079C-s003 [file DT-051-D2DT02079C-s003.gif]
